# Multi-Response Optimization of Controlled Germination Conditions Enhances Functional and Bioactive Properties of Andean *Kañiwa* (*Chenopodium pallidicaule* Aellen cv. Cupi)

**DOI:** 10.3390/molecules31172947

**Published:** 2026-08-22

**Authors:** Hans Himbler Minchán-Velayarce, Edward Florencio Aurora-Vigo, Gabriel Júnio Silva Souza, Suny Chen Luera-Quiñones, Jorge Coronado-Olano, Luz María Paucar-Menacho, Nathália de Andrade Neves, Tarcisio Michael Ferreira Soares de Oliveira, Raniere Ribeiro Lima, Vivian Machado Benassi, Julio Vidaurre-Ruiz, Marcio Schmiele

**Affiliations:** 1 Programa de Doctorado en Ingeniería Agroindustrial Mención Transformación Avanzada de Granos y Tubérculos Andinos, Universidad Nacional del Santa, Nuevo Chimbote 02712, Peru; 2024818002@uns.edu.pe (E.F.A.-V.); 2024818018@uns.edu.pe (S.C.L.-Q.); 2024818035@uns.edu.pe (J.C.-O.); 2 Grupo de Investigación en Compuestos Bioactivos a Partir de Matrices Alimentarias y Biológicas—COMBALBI, Universidad Nacional de Jaén, Car. Jaén—San Ignacio Km. 24 Sec., Jaén 06801, Peru; 3 Laboratório Integrado de Cereais e Lipídeos (LICEL), Instituto de Ciência e Tecnologia (ICT), Universidade Federal dos Vales do Jequitinhonha e Mucuri (UFVJM), Diamantina 39100-000, Brazil; junio.gabriel@ufvjm.edu.br; 4 Departamento Académico de Agroindustria y Agronomía, Facultad de Ingeniería, Universidad Nacional del Santa, Chimbote 02712, Peru; luzpaucar@uns.edu.pe; 5 Laboratório de Pesquisa em Bioativos de Plantas (FITOLAB), Departamento de Agronomia, Faculdade de Ciências Agrárias (FCA), Universidade Federal dos Vales do Jequitinhonha e Mucuri (UFVJM), Diamantina 39100-000, Brazil; nathalia.neves@ufvjm.edu.br; 6 Laboratório de Micologia, Enzimologia e Desenvolvimento de Produtos (LMEDP), Instituto de Ciência e Tecnologia (ICT), Universidade Federal dos Vales do Jequitinhonha e Mucuri (UFVJM), Diamantina 39100-000, Brazil; tarcisio.michael@ufvjm.edu.br (T.M.F.S.d.O.); raniere.ribeiro@ufvjm.edu.br (R.R.L.); vivian.benassi@ict.ufvjm.edu.br (V.M.B.); 7 Centro de Investigación e Innovación en Productos Derivados de Cultivos Andinos CIINCA, Universidad Nacional Agraria La Molina, Lima 15024, Peru; vidaurrejm@lamolina.edu.pe

**Keywords:** response surface methodology, GABA, bioactive compounds, ORAC, FRAP, pseudocereal, sprouting, gluten-free ingredient

## Abstract

*Kañiwa* (*Chenopodium pallidicaule* Aellen) is an Andean pseudocereal rich in protein and phenolic compounds, whose bioactive potential under controlled germination remains unexplored through simultaneous metabolite optimization. Germination conditions of *kañiwa* cv. Cupi were optimized using a Central Composite Design (CCD) and the Derringer–Suich desirability function, with time (24–96 h) and temperature (18–30 °C) as independent variables, and *γ*-aminobutyric acid (GABA), total soluble phenolic compounds (TSPC), ORAC, FRAP, and protein content as responses. Five second-order polynomial models yielded adjusted R^2^ values of 0.703–0.880 with non-significant lack of fit (*p* > 0.050). Germination time was the dominant factor for GABA, TSPC, ORAC, and FRAP, while temperature exerted the predominant negative quadratic effect on protein content. Optimal conditions (96 h, 24 °C; D = 0.845) produced experimental increases of 1710.30% in GABA (26.12 mg GABA·100 g^−1^), 117.10% in TSPC (926.9 mg GAE·100 g^−1^), 261.40% in ORAC (51,756.0 µmol TE·100 g^−1^), 244.10% in FRAP (1971.2 µmol TE·100 g^−1^), and 42.34% in protein (16.05%) relative to ungerminated seeds. The GABA value constitutes the first report of this non-protein amino acid in germinated *kañiwa* under optimized conditions. Complementary analyses demonstrated that optimized germination markedly increased total reducing sugars and free glucose while decreasing amylolytic and glucoamylase activities and increasing lipase activity, confirming the extensive mobilization of carbohydrate reserves and the metabolic reprogramming associated with the germination process. These findings position *kañiwa* as a high-value functional ingredient for the Andean food industry.

## 1. Introduction

*Kañiwa* (*Chenopodium pallidicaule* Aellen) is an Andean pseudocereal cultivated between 3800 and 4400 m.a.s.l. in Peru and Bolivia, where it is recognized for its remarkable tolerance to frost, drought, and saline soils [1,2,3,4]. Nutritionally, *kañiwa* contains approximately 12.8% protein and exhibits a high-quality amino acid profile comparable to that of casein, with no limiting amino acids, and a high concentration of glutamic acid (16.91 g·100 g^−1^ of protein), the main precursor of *γ*-aminobutyric acid (GABA) biosynthesis during germination [2,3]. In addition to its protein quality, *kañiwa* is recognized as a source of phenolic compounds and antioxidant constituents, with ungerminated seeds containing approximately 85.7 mg GAE·100 g^−1^ of total phenolics and 150,980 µg TE·100 g^−1^ of antioxidant capacity measured by DPPH assay [4]. However, the reported concentrations of phenolic compounds vary considerably among studies due to differences in extraction procedures and units of expression. For example, the value reported by Repo-Carrasco-Valencia et al. [1] was obtained using a simple methanolic extraction, whereas sequential methanol–acetone extraction systems, such as that employed in the present study, have been shown to improve the recovery of soluble phenolic [5].

Germination triggers a series of biochemical and physiological processes mediated by the activation of endogenous enzymes, resulting in the synthesis, bioconversion and accumulation of bioactive metabolites [6,7,8,9,10,11,12]. Previous studies have demonstrated high germination efficiencies in Andean grains and pseudocereals, with germination rates reaching 95.3% in quinoa germinated at 20 °C for 48 h and 89.7% in amaranth germinated at 25 °C for 72 h [12,13,14,15,16]. Beyond promoting seed viability, germination has been reported to enhance the nutritional and functional properties of pseudocereals. In *kañiwa* germinated at 20 °C for 72 h, total phenolics increased by 17% and TEAC antioxidant capacity rose 27%, from 70.9 to 100.9 µmol TE·g^−1^ [1,17,18]. These findings suggest that controlled germination may represent an effective strategy to enhance the bioactive potential of *kañiwa* and expand its application as a functional food ingredient.

*γ*-Aminobutyric acid (GABA) is synthesized by decarboxylation of glutamic acid via glutamate decarboxylase (GAD), a pyridoxal 5′-phosphate-dependent enzyme whose activity increases markedly during germination [2,6,7,13,19,20,21]. GABA has attracted considerable interest in food science due to its hypotensive, anxiolytic, neuroprotective, and lipid metabolism-modulating properties, making germinated grains and pseudocereals promising ingredients for the development of functional foods [6,8,13,17,21,22]. In soybean germinated at 25 °C for 42 h, GABA content increased by 61.7%, while in *kiwicha* germinated at 26 °C for 63 h, GABA content reached 75.7 mg·100 g^−1^, representing a 29-fold increase compared with the ungerminated grain [9,13,14,15]. Under identical conditions, purple maize accumulated 74.4 mg GABA·100 g^−1^ [9,20]. Despite the recognized nutritional potential of *kañiwa* and its naturally high glutamic acid content, no studies have reported the optimization of germination conditions to maximize GABA in *kañiwa* [2,13].

Phenolic compounds also increased during germination as a consequence of phenylalanine ammonia-lyase (PAL) activation, which stimulates the phenylpropanoid pathway and promotes the biosynthesis of new phenolic metabolites [6,17]. Simultaneously, endogenous hydrolytic enzymes release bound phenolics through the cleavage of ester linkages between phenolic compounds and cell wall polysaccharides [15,17,23,24]. In quinoa germinated at 20 °C for 42 h, TSPC increased from 271.0 to 489.8 mg GAE·100 g^−1^, representing an 80.7% (1.8-fold) increase [14]. Likewise, in *kiwicha* germinated at 26 °C for 63 h, TSPC increased by 347%, from 86.22 to 385.4 mg GAE·100 g^−1^, accompanied by the formation of bioactive compounds such as 4-*O*-caffeoylquinic acid (0 to 880 µg·g^−1^) and 4-*O*-feruloylquinic acid (0 to 112 µg·g^−1^), and the enhancement of quercetin 3-*O*-rutinoside (from 2.15 to 13.7 µg·g^−1^) [23]. In amaranth germinated at 30 °C for 78 h, total phenolics increased by 829%, from approximately 26.7 to 247.6 mg GAE·100 g^−1^ [15]. These findings demonstrate that germination can substantially enhance the phytochemical profile of pseudocereals; however, information regarding the simultaneous optimization of phenolic accumulation in *kañiwa* remains scarce.

Antioxidant capacity is closely associated with the accumulation of phenolic compounds and GABA during germination [22]. In *kiwicha* germinated at 26 °C for 63 h, ORAC values reached 10,090 µmol TE·100 g^−1^ [23], whereas red quinoa germinated at 17 °C for six days exhibited a 178.9% increase in total soluble phenolic compounds and a calculated 69.9% increase in its total antioxidant activity, reaching 29,560 µmol TE·100 g^−1^ [25]. For *kañiwa* germinated at 20 °C for 72 h, total antioxidant capacity measured by direct ABTS assay increased by 42.3%, from 7095 to 10,094 µmol TE·g^−1^ [1].

Response Surface Methodology (RSM) enables process optimization through strategic designed experimental, reducing the number of trials while accurately describing the combined effects of process variables and their interactions [9,19,26]. In soybean BRS 133, a rotatable central composite design established that 42 h at 25 °C maximized lunasin content (+61.7%), while reducing lectin (−58.7%), and lipoxygenase activity (−70.0%) [27]. For millet, RSM established 28 °C and 54 h as optimal conditions for hydroxyl radical inhibition, achieving 60.38% inhibition with R^2^ = 0.9847 [28]. For millet fiber, conditions of 82.2 °C, 5.1 h, and a 1:20 ratio yielded an extraction of 3.65% [7]. These studies demonstrate the effectiveness of RSM for optimizing germination-driven biochemical responses. However, no validated protocol has been reported for *kañiwa* focusing on the simultaneous enhancement of GABA, phenolic compounds, and antioxidant capacity.

Prior evidence on *kañiwa* germination is restricted to a single study conducted at a fixed temperature of 20 °C, which did not vary temperature as a factor, did not fit a predictive model, and did not evaluate GABA or protein content [1]. A single-factor design cannot locate the optimum when the two factors interact, a situation confirmed in the present study: the time × temperature interaction was significant for TSPC (*p* = 0.039) and approached significance for GABA (*p* = 0.058). Furthermore, simultaneous multi-response optimization differs fundamentally from single-response optimization, since the surfaces of different bioactive responses are not concordant and an optimum identified for one response does not necessarily coincide with the optimum for another. This study therefore addresses three specific gaps: the absence of any quantification of GABA in *kañiwa* under controlled germination, the absence of a predictive model for the joint effect of germination time and temperature in this species, and the absence of a simultaneous multi-response optimization whose individual optima do not coincide. To address these gaps, germination conditions of *kañiwa* cv. Cupi were optimized using a Rotatable Central Composite Design and the Derringer–Suich desirability function to simultaneously maximize GABA, TSPC, antioxidant capacity (ORAC and FRAP) and protein content.

## 2. Materials and Methods

### 2.1. Reagents

The reagents used in this study included Folin–Ciocalteu 2 N reagent, gallic acid, 6-hydroxy-2,5,7,8-tetramethylchroman-2-carboxylic acid (Trolox), fluorescein, 2,2′-azobis(2-amidinopropane) dihydrochloride (AAPH), 2,4,6-tri(2-pyridyl)-s-triazine (TPTZ), iron(III) chloride hexahydrate (FeCl_3_·6H_2_O), and ethylenediaminetetraacetic acid (EDTA, calibration standard for elemental analysis), all acquired from Sigma-Aldrich Co. (St. Louis, MO, USA). Methanol (HPLC grade), acetonitrile (HPLC grade), acetone, *γ*-aminobutyric acid (GABA, purity > 99%), and sodium acetate trihydrate were acquired from Merck KGaA (Darmstadt, Germany). Sodium carbonate, sodium hydroxide, and sodium hypochlorite were acquired from J.T. Baker (Phillipsburg, NJ, USA). All reagents were of analytical grade and used without further purification.

### 2.2. Plant Material

**Figure 1:**
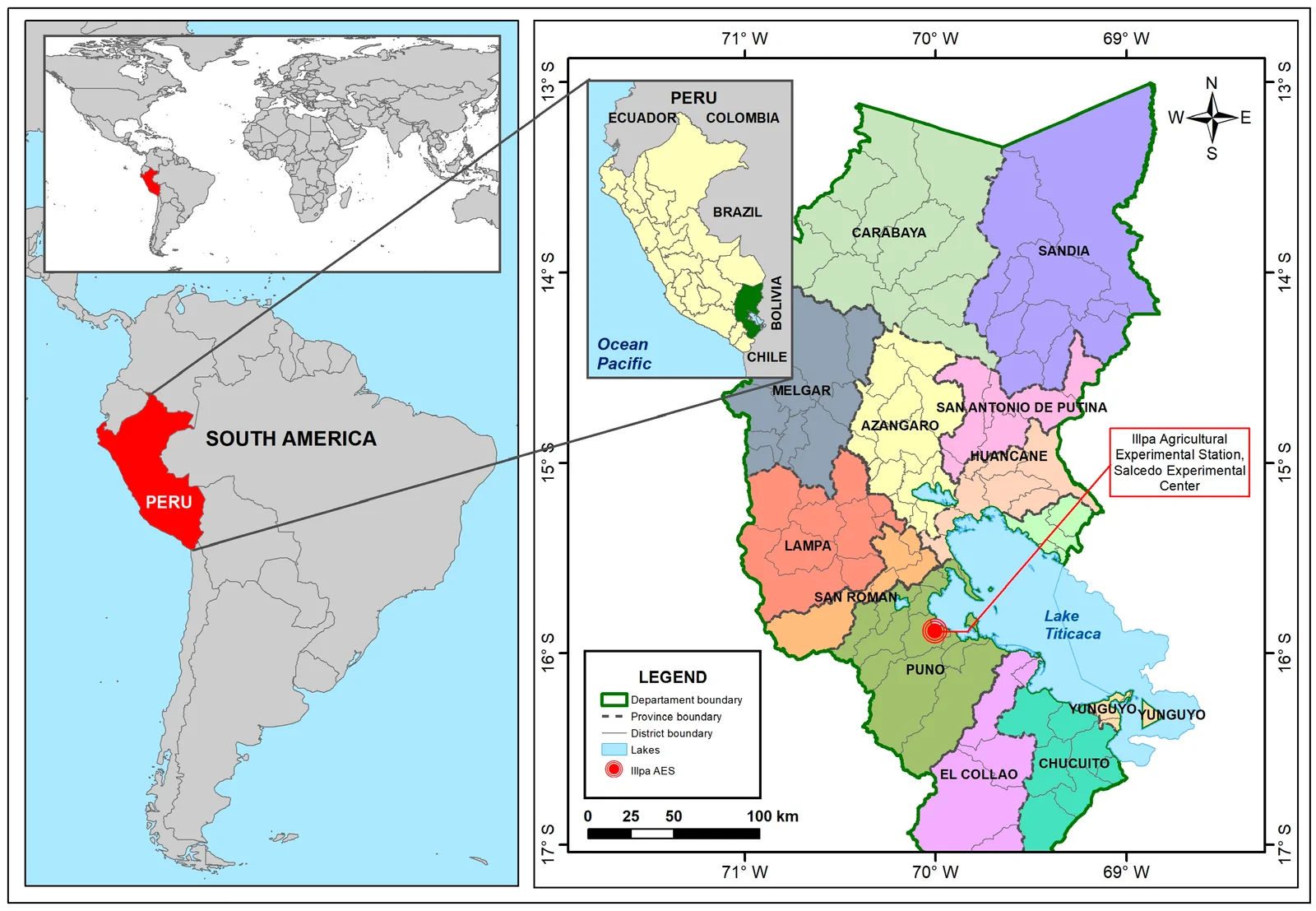
Location of the Illpa Agricultural Experimental Station (INIA–EEA Illpa), Centro Experimental Salcedo, Department of Puno, Peru (15°52′56.2″ S, 70°00′08.9″ W; 3844 m a.s.l.). Map produced in ArcGIS Desktop 10.5 (Esri Inc., Redlands, CA, USA). Administrative and provincial boundaries: Instituto Geográfico Nacional del Perú (IGN); projection: Universal Transverse Mercator (UTM) Zone 19S, datum WGS 84. INIA: Instituto Nacional de Innovación Agraria; EEA: Estación Experimental Agraria.

Seeds of the *kañiwa* variety Cupi were obtained from the Instituto Nacional de Innovación Agraria (INIA), Estación Experimental Agraria Illpa, Centro Experimental Salcedo, Puno, Peru (15°52′56.2″ S, 70°00′08.9″ W; −15.882282, −70.002478, approximately 3844 m a.s.l.) (Figure 1). Seeds were manually sorted to remove impurities, damaged grains, and foreign material, and evaluated for viability >80% and morphological uniformity. Selected seeds were stored between 4–8 °C under dry conditions until use.

### 2.3. Seed Preparation for Germination

Seeds were prepared following a standardized protocol previously described in the literature [14,23]. Briefly, 100 g of seeds were rinsed with tap water and disinfected with 0.1% (*w*/*v*) sodium hypochlorite solution at a seed-to-solution ratio of 1:5 for 30 min at room temperature. The disinfected seeds were subsequently drained and rinsed five times with tap water until a neutral pH was achieved. Hydration was then performed by soaking the seeds in tap water at a 1:5 (*w*/*v*) ratio for 8 h at room temperature. After soaking, the excess water was drained, and the hydrated seeds were immediately transferred to the germination system.

### 2.4. Germination System

The germination system consisted of rectangular plastic trays (305 × 195 × 60 mm) fitted with perforated stainless-steel grids (230 × 138 × 50 mm). Slow-flow filter paper sheets (60 × 60 cm) were cut into two sizes: rectangular size A (230 × 138 mm), placed directly over the grid, and square sheet B (230 × 230 mm), used to cover the hydrated seeds.

Each tray was filled to approximately two-thirds of its capacity with tap water. The stainless-steel grid was lined with filter paper, previously moistened with tap water, after which the hydrated seeds were uniformly distributed in a thin layer over the surface. The seeds were then covered with filter paper B, ensuring that both ends were immersed in the water reservoir to ensure a continuous moisture supply by capillary action throughout the germination period.

The assembled trays were transferred to a BJPX-HT400BII grain germinator chamber (BIOBASE, Jinan, China) equipped with a water circulation system that maintained the relative humidity at approximately 80%. Germination temperature and time were adjusted according to the experimental design, and all treatments were conducted under complete darkness to avoid light-induced metabolic responses.

### 2.5. Drying and Storage

At the end of the programmed germination period, germinated seeds were removed and transferred to stainless-steel trays. Drying was carried out in an SBT-10X10, JP 001 0113 tray dryer (Torrh, Junin, Peru) at 40–45 °C for 48 h until the moisture content reached 5–7%. This relatively mild drying condition was selected to minimize the degradation of thermolabile bioactive compounds generated during germination. The dried material was ground using a stainless-steel high-speed knife mill/grinder, ASIN B0BRR6J94K (PURRL, Ningbo, China) and vacuum-packed using an Auster vacuum sealer (Besser Vacuum, Dignano, Italy) in nylon poly bags and stored under refrigeration in a GC-J237JSPN LG Inverter Linear Compressor refrigerator (LG Electronics, Seoul, Republic of Korea) between 4–8 °C.

### 2.6. Experimental Design

#### 2.6.1. Independent Variables

A Central Composite Design (CCD) with two factors was employed, totaling 12 experimental units. The independent variables evaluated were X_1_: germination time (h) and X_2_: germination temperature (°C). The coded and real levels are presented in Table 1.

Variables were coded according to Equation (1) [29,30]:

(1) $$x_{i}=\frac{X_{i}-X_{0}}{\Delta x_{i}}$$

where *x_i_* is the coded value of the independent variable, *X_i_* is the real value of the independent variable, *X*_0_ is the real value at the central point, and Δ*x_i_* is the step change value.

#### 2.6.2. Experimental Design Matrix

The CCD experimental matrix is presented in Table 2.

#### 2.6.3. Response Variables

The response variables evaluated were: *γ*-aminobutyric acid (GABA) content expressed as mg GABA·100 g^−1^; total soluble phenolic compounds (TSPC) expressed as mg gallic acid equivalents (GAE)·100 g^−1^; antioxidant capacity determined by two complementary methods, oxygen radical absorbance capacity (ORAC) and ferric reducing antioxidant power (FRAP), both were expressed as µmol Trolox equivalents (TE)·100 g^−1^; and protein content expressed as percentage.

### 2.7. Determination of GABA

*γ*-Aminobutyric acid content was determined by reverse-phase HPLC after pre-column derivatization, following [31] with modifications by [32], under the same conditions previously described for germinated cereals and adapted to the present matrix. Briefly, GABA extraction was assisted by low-frequency ultrasound using an ultrasonic bath (CBU/100/3LDG, Planatec, São Paulo, Brazil) operating at 40 kHz and 100 W. In duplicate, ground samples (1.0 g) were mixed with 4.5 mL of 75% ethanol (*v*/*v*) in 15-mL Falcon tubes and vortexed for 1 min using a NA 3600 vortex mixer (Norte Científica, Araraquara, Brazil). The mixture was then sonicated for 10 min at room temperature (20 °C), followed by centrifugation at 3200× *g* for 5 min in a Sorvall ST8 centrifuge (Thermo Fisher Scientific, Waltham, MA, USA). The supernatant was filtered through qualitative filter paper (Unifil, 9 cm diameter, 80 g·m^−2^) and collected in a 10-mL volumetric flask. The extraction procedure was repeated once, and the final volume was adjusted to 10 mL with the extraction solvent.

For derivatization, 100 µL of extract was mixed with 200 µL of 10 mM dansyl chloride solution prepared in HPLC-grade acetonitrile and 200 µL of 5 M sodium carbonate buffer (pH 10). The reaction mixture was sonicated for 3 min and subsequently incubated at 65 °C for 25 min in a static SL-150 water bath (Solab, Piracicaba, Brazil). After derivatization, samples were centrifuged at 10,000× *g* for 15 min at 4 °C using a SL-5GR refrigerated centrifuge (Spinlab, Ribeirão Preto, Brazil).

Chromatographic analysis was performed using a 1260 Infinity HPLC system (Agilent Technologies, Santa Clara, CA, USA) equipped with a quaternary pump and a diode-array detector (DAD G1314B). Separation was achieved on a Zorbax Eclipse C18 column (Agilent Technologies, Santa Clara, CA, USA) (4.6 × 100 mm, 3.5 µm particle size). The mobile phase consisted of 0.05 M sodium phosphate buffer (pH 7.2) as solvent A and acetonitrile as solvent B, delivered at a flow rate of 1.0 mL·min^−1^. The gradient program was as follows: 14–20% B (0–7 min), 20–22% B (7–10 min), and 22–25% B (10–15 min). Subsequently, solvent B was increased to 85% and maintained for 3 min, followed by re-equilibration from 85 to 14% B between 25 and 26 min. The injection volume was 10 µL, and detection was performed at 254 nm [31]. Quantification was carried out using external calibration with a *γ*-aminobutyric acid analytical standard (Merck, purity > 99%). The calibration curve was prepared in the concentration range of 0–184 mg·L^−1^ and showed excellent linearity (y = 29.245x + 30.702; R^2^ = 0.9988). Results were expressed as mg GABA·100 g^−1^ (d.b.).

### 2.8. Extraction of Total Soluble Phenolic Compounds (TSPC)

The extraction solution for total soluble phenolic compounds in *kañiwa* was previously optimized by our research group [33] using a Simplex-Lattice mixture design, yielding an acidified hydroalcoholic-acetonic solution composed of water (1.27%, *v*/*v*), methanol (2.17%, *v*/*v*), ethanol (43.28%, *v*/*v*), and acetone (53.30%, *v*/*v*), acidified with 0.1 mL of concentrated HCl per 5 mL of solvent. Briefly, 200 ± 2 mg of ground sample was weighed into a 2 mL microcentrifuge tube and subjected to eight sequential extraction cycles performed on the same residue. In each cycle, 1.5 mL of extraction solvent was added to the sample and vortexed for 30 s, followed by sonication (40 kHz, 100 W, 5 min, 20 ± 2 °C) and centrifugation at 10,000 rpm for 10 min at room temperature using a MiniSpin plus microcentrifuge equipped with an *F*-45-12-11 fixed-angle rotor (Eppendorf, Hamburg, Germany). The supernatant was carefully recovered and brought to a final volume of 2.0 mL with extraction solvent, while the residual pellet was re-extracted with 1.5 mL of fresh solvent under identical conditions for the subsequent cycle, without discarding the pellet between cycles. According to the validated extraction model, 95% recovery of total soluble phenolic compounds was reached at the seventh cycle.

### 2.9. Quantification of Total Soluble Phenolic Compounds

Total soluble phenolic compounds (TSPC) was determined using the Folin–Ciocalteu colorimetric assay, following [5] a micro-scale protocol adapted in our laboratory. An aliquot of the diluted extract (23 µL) was mixed with 57 µL of Folin–Ciocalteu reagent (1 N). After 5 min, 230 µL of 15% (*w*/*v*) sodium carbonate solution was added, and the reaction volume was adjusted to 1000 µL with distilled water (690 µL). The mixture was vortexed thoroughly and incubated for 60 min at room temperature in the dark. Absorbance was measured at 765 nm using a Synergy H1 Hybrid Multi-Mode Microplate Reader (BioTek Instruments Inc., Winooski, VT, USA). Gallic acid (50–250 mg L^−1^; five-point curve) was used as an external standard to construct the calibration curve (Y = 0.00156X − 0.0169; R^2^ = 0.9955), and results were expressed as mg GAE·100 g^−1^ (d.b.). All determinations were performed in triplicate.

### 2.10. Determination of Antioxidant Capacity

Antioxidant capacity of the phenolic extracts was evaluated by two assays: oxygen radical absorbance capacity (ORAC) and ferric reducing antioxidant power (FRAP). Results of all assays were expressed as µmol Trolox equivalents (TE)·100 g^−1^ (d.b.).

#### 2.10.1. ORAC Assay

ORAC was determined according to [34] with minor modifications, using a fluorescence microplate reader. Sample extracts were diluted (1:10–1:50) in 75 mM sodium phosphate buffer (pH 7.4). In black 96-well plates, 25 µL of diluted sample or Trolox standard (0–200 µM) was mixed with 150 µL of fluorescein solution (final concentration ≈ 70 nM) and pre-incubated at 37 °C for 15 min. The reaction was initiated by adding 25 µL of AAPH solution and fluorescence (excitation 485 nm, emission 528 nm) was recorded every minute for 105 min. The area under the fluorescence decay curve (AUC) was calculated for each well, and net AUC values (sample or standard minus blank) were used to construct the Trolox calibration curve (0–160 µM, *n* = 8 points), expressed as Y = 0.106X + 1.450 (R^2^ = 0.9937). Results were expressed as µmol TE·100 g^−1^ (d.b.), applying a confirmed dilution factor of 1/10 (DF = 10). Analyses were performed in triplicate.

#### 2.10.2. FRAP Assay

Ferric reducing antioxidant power was measured according to [35]. The FRAP reagent was freshly prepared by mixing acetate buffer (300 mM, pH 3.6), TPTZ solution (10 mM in HCl, pH ≈ 1.4), and FeCl_3_·6H_2_O solution (20 mM) in a 10:1:1 (*v*/*v*/*v*) ratio and equilibrating at 37 °C before use. In 96-well plates, 20 µL of diluted extract or Trolox standard (0–600 µM) was added to 180 µL of FRAP reagent and incubated at 37 °C. After the development time specified in the standardized protocol (4–10 min), absorbance was recorded at 593 nm. Trolox (25–600 µM; seven-point curve) was used as an external standard to construct the calibration curve (Y = 0.00332x − 0.0144; R^2^ = 0.9998), and results were expressed as µmol TE·100 g^−1^ (d.b.). Measurements were carried out in triplicate.

### 2.11. Determination of Protein Content

Total protein content was determined by Dumas combustion, in accordance with AOAC official method 990.03 [36], using a LECO CHNS-628 elemental analyzer (LECO Corporation, St. Joseph, MI, USA). Samples were previously homogenized, dried to constant weight, and ground to approximately 149 µm particle size. For analysis, 100 mg of sample was weighed into tin capsules, sealed, and subjected to complete combustion in an oxygen atmosphere using helium as carrier gas. Released nitrogen was converted to N_2_ by reduction over a copper column, following removal of H_2_O and CO_2_ by specific absorbents, and quantified by thermal conductivity detection. Instrumental calibration was performed with EDTA as the primary reference standard, with periodic blank verification. Total nitrogen content was converted to crude protein using the general factor of 6.25. All determinations were carried out in triplicate.

### 2.12. Multi-Objective Optimization

Simultaneous optimization of the five response variables (TSPC, ORAC, FRAP, GABA, and protein content) was performed using the [37] desirability function implemented in the *Prediction & Profiling* module of Statistica v14.0.0.15. The desirability function transforms each response *Yᴵ* into a partial desirability *dᴵ* ∈ [0, 1] according to Low (d = 0), Medium (d = 0.50), and High (d = 1.0) thresholds, and calculates the overall desirability *D* as the geometric mean of the *n* individual desirabilities [26,38,39] (Equation (2)):

(2) $$D={\left(\overset{n}{\underset{i=1}{\prod }}d_{i}\right)}^{1/n}$$

Simultaneous maximization of all response variables was set as the optimization criterion, with symmetric linear curvature (s = t = 1.0) for each partial desirability function. The optimum point was located using the *Use general function optimization* option with quadratic fitting surface [29].

### 2.13. Validation Experiments

To verify the predictive validity of the RSM models, confirmation experiments were conducted in triplicate under the optimal conditions identified by the desirability function. The relative deviation between predicted and experimental values was calculated for each response variable as a complementary indicator of the predictive capability of the RSM models, in conjunction with the goodness-of-fit statistics (R^2^ adj, *p*-value, lack-of-fit) [23,26,29].

### 2.14. Determination of Reducing Sugar Content and Endogenous Enzyme Activities

Ungerminated *kañiwa* and the sample germinated under the optimized conditions were evaluated for reducing sugar content and the activities of amylolytic enzymes, glucoamylase, lipase, and protease. The determination of reducing sugars was included as an indirect indicator of starch hydrolysis during the 96-h germination period, providing complementary information on reserve carbohydrate mobilization under the optimized germination conditions. Since intermediate germination stages were not evaluated, the reducing sugar content reflects the cumulative extent of starch degradation achieved at the end of the germination process rather than the kinetics of hydrolysis throughout germination.

#### 2.14.1. Preparation of the Crude Enzymatic Extract

The crude enzymatic extract was obtained from both ungerminated *kañiwa* seeds and seeds germinated under the optimized conditions. Briefly, 1.0 g of the sample was weighed into a 15 mL Falcon tube, followed by the addition of 10 mL of 100 mM sodium acetate buffer (pH 5.0). The mixtures were subjected to ultrasonic-assisted extraction in an ultrasonic bath (100 W, 40 kHz) for 10 min at 20 °C. Subsequently, the samples were centrifuged at 2000× *g* for 10 min at 20 °C, and the supernatant was collected into a 10 mL volumetric flask. The extraction procedure was repeated exhaustively for three consecutive cycles, as previously established in preliminary experiments, and the combined extracts were adjusted to a final volume of 10 mL with the extraction buffer. The extraction pH (100 mM sodium acetate buffer, pH 5.0) was chosen to obtain a single, common crude extract for all four enzyme activities, thereby eliminating extract-to-extract variability between ungerminated and germinated samples. Each activity was subsequently measured under its own reaction conditions and buffer: amylolytic activity in 100 mM sodium acetate buffer (pH 5.0), glucoamylase activity in 100 mM sodium citrate buffer (pH 6.0), and lipase and protease activities in 100 mM sodium acetate buffer (pH 6.0), all at 50 °C. Mildly acidic buffers are routinely used to recover seed hydrolases, which are predominantly associated with protein bodies and vacuoles; solubilization was ensured by three exhaustive ultrasound-assisted extraction cycles. Enzymes with unfavorable solubility at pH 5.0, particularly serine-type proteases active above pH 7.0, may be under-recovered under these conditions, which is acknowledged as a limitation of the enzymatic characterization (Section 3.9).

#### 2.14.2. Determination of Total Reducing Sugars

Total reducing sugars were quantified in triplicate using the 3,5-dinitrosalicylic acid (DNS) colorimetric method described by [40]. Briefly, 50 μL of the crude enzymatic extract was transferred to a microtube and heated in a boiling water bath (~94 °C, accounting for the altitude of Diamantina, Brazil, at approximately 1200 m above sea level). Subsequently, 50 μL of DNS reagent was added, and the reaction mixture was boiled for 5 min. After cooling to room temperature (~20 °C), 500 μL of distilled water was added to each tube. Absorbance was measured at 540 nm using an LMR-96 microplate spectrophotometer (Loccus, Cotia, Brazil). A reagent blank was prepared by replacing the enzymatic extract with distilled water. An eleven-point glucose calibration curve (0 to 5 mg·mL^−1^; y = 0.800x; r = 0.9828) was used for quantification, and the results were expressed as mg of reducing sugars per g of sample (d.b.).

#### 2.14.3. Determination of Free Glucose

Free glucose was determined using the glucose oxidase–peroxidase (GOPOD, Bioclin, Belo Horizonte, Brazil) enzymatic colorimetric method described in [41]. Before analysis, a 500 μL aliquot of the crude enzymatic extract was heated in a boiling water bath for 5 min to inactivate endogenous enzymes and subsequently cooled to room temperature. An aliquot of 10 μL of the treated extract was then mixed with 1.0 mL of GOPOD reagent, and the color reaction was allowed to proceed for 20 min at room temperature. The reagent blank (time zero) was prepared by replacing the enzymatic extract with distilled water. Absorbance was measured at 510 nm using a UV-M51 spectrophotometer (Bel Photonics, Monza, Italy). Free glucose concentration was determined from a glucose standard curve and expressed as mg of glucose per g of sample (d.b.).

#### 2.14.4. Determination of Amylolytic Activity

Amylolytic activity was determined using 1% (*w*/*v*) soluble starch (Cinética^®^, Itapevi, Brazil) prepared in 100 mM sodium acetate buffer (pH 5.0) as the substrate. Enzyme activity was quantified by measuring the reducing sugars released during the enzymatic hydrolysis of starch using the 3,5-dinitrosalicylic acid (DNS) colorimetric method described by Miller [40]. The enzymatic reaction was initiated by mixing 200 μL of the starch substrate with 200 μL of the crude enzymatic extract, followed by incubation in a thermostatic water bath (SL-150, Solab, Piracicaba, Brazil) at 50 °C for 10 min. After incubation, a 50 μL aliquot of the reaction mixture was transferred to a tube containing 50 μL of DNS reagent to terminate the reaction. The tubes were then heated in a boiling water bath for 5 min, cooled to room temperature, and diluted with 500 μL of distilled water. The reaction blank (time zero) was prepared by immediately transferring a 50 μL aliquot of the reaction mixture to the DNS reagent before incubation, thereby accounting for the initial concentration of reducing sugars present in the extract. Absorbance was measured at 540 nm using an LMR-96 microplate spectrophotometer (Loccus, Cotia, Brazil).

Reducing sugar concentration was determined using an 11-point glucose standard curve (0–5.0 mg·mL^−1^; y = 0.800x; r = 0.9828). All analyses were performed in triplicate. One unit (U) of amylolytic activity was defined as the amount of enzyme required to release 1 μmol of reducing sugars per minute under the assay conditions, and the results were expressed as U·g^−1^ of sample (d.b.).

#### 2.14.5. Determination of Glucoamylase Activity

Glucoamylase activity was determined using the glucose oxidase–peroxidase (GOPOD) enzymatic-colorimetric method described by Bergmeyer and Bernt [41], employing a commercial glucose oxidase/peroxidase reagent kit (Bioclin^®^, Belo Horizonte, Brazil). The reaction mixture consisted of 200 μL of crude enzymatic extract and 200 μL of 1% (*w*/*v*) maltose prepared in 100 mM sodium citrate buffer (pH 6.0). The reaction was incubated in a thermostatic water bath at 50 °C for 10 min. After incubation, a 10 μL aliquot of the reaction mixture was withdrawn, heated in a boiling water bath for 5 min to terminate the enzymatic reaction, and cooled to room temperature. Subsequently, 1.0 mL of GOPOD reagent was added, and the mixture was incubated for 20 min at room temperature to allow color development.

The reaction blank (time zero) was prepared by boiling the crude enzymatic extract before the addition of the maltose substrate, thereby inactivating the enzyme before the reaction. After substrate addition, 1.0 mL of GOPOD reagent was added, and the mixture was incubated for 20 min under the same conditions as the samples. Absorbance was measured at 510 nm using a UV-M51 spectrophotometer (Bel Photonics, Monza, Italy).

Glucose quantification was performed using the glucose standard solution (100 mg·dL^−1^) supplied with the commercial kit, following the manufacturer’s instructions. One unit (U) of glucoamylase activity was defined as the amount of enzyme required to produce 1 μmol of glucose per minute under the assay conditions, and the results were expressed as U·g^−1^ of sample (d.b.).

#### 2.14.6. Determination of Lipase Activity

Lipase activity was determined using *p*-nitrophenyl palmitate (*p*-NPP) as the substrate according to the method described by [42]. Two stock solutions were prepared before the assay. Solution I consisted of 0.01 g of gum arabic (Sigma-Aldrich Co., St. Louis, MO, USA), 18 mL of 100 mM sodium acetate buffer (pH 6.0) (Sigma-Aldrich Co., St. Louis, MO, USA), and 50 μL of Triton X-100 (Synth, Diadema, Brazil), which were homogenized under magnetic stirring for 25 min. Solution II consisted of 0.03 g of *p*-nitrophenyl palmitate dissolved in 10 mL of isopropanol and homogenized under magnetic stirring for 5 min. Immediately before the assay, Solutions I and II were mixed and stirred for an additional 10 min to obtain the reaction medium.

The enzymatic reaction was initiated by mixing 200 μL of the crude enzymatic extract with 200 μL of the reaction medium, followed by incubation in a thermostatic water bath at 50 °C for 10 min. The reaction was terminated by adding 400 μL of saturated sodium tetraborate solution. A blank was prepared using boiled crude enzymatic extract to account for non-enzymatic hydrolysis of the substrate.

A standard curve was prepared using *p*-nitrophenol (0–1.0 μmol·mL^−1^; y = 5.17072x; r = 0.9996). Absorbance was measured at 405 nm using a microplate spectrophotometer (LMR-96, Loccus, Cotia, Brazil). All analyses were performed in triplicate. One unit (U) of lipase activity was defined as the amount of enzyme required to release 1 μmol of *p*-nitrophenol per minute under the assay conditions, and the results were expressed as U·g^−1^ of sample (d.b.).

#### 2.14.7. Determination of Protease Activity

Protease activity was determined in triplicate using 1% (*w*/*v*) casein (Sigma-Aldrich, St. Louis, MO, USA) prepared in 100 mM sodium acetate buffer (pH 6.0) [43], with modifications. The enzymatic reaction was carried out in 1.5 mL microcentrifuge tubes by incubating 300 μL of the substrate solution with 300 μL of the crude enzymatic extract in a thermostatic water bath at 50 °C for 10 min. The reaction was terminated by adding 600 μL of 20% (*w*/*v*) trichloroacetic acid (TCA) (Synth, Diadema, Brazil).

The reaction mixtures were then kept in an ice bath for 30 min to allow precipitation of undigested proteins, followed by centrifugation at 10,000× *g* for 10 min. The reaction blank was prepared using boiled crude enzymatic extract, which was immediately mixed with 300 μL of the casein substrate and 600 μL of 20% TCA to account for non-enzymatic absorbance. An aliquot of the clear supernatant was transferred to a quartz cuvette before absorbance measurement. The absorbance of the supernatant was measured at 280 nm using a UV-M51 spectrophotometer (Bel Photonics, Monza, Italy), according to Batista [44].

A standard curve was prepared using *L*-tyrosine (0–0.3 mg·mL^−1^; y = 1.629x; r = 0.9997). One unit (U) of protease activity was defined as the amount of enzyme required to release 1 μmol of tyrosine equivalents during casein hydrolysis per minute under the assay conditions, and the results were expressed as U·g^−1^ of sample (d.b.).

### 2.15. Predictive Mathematical Model

The response surface model (Equation (3)) was fitted using a second-order polynomial equation [39]:

(3) $$Y=\beta_{0}+\sum \beta_{i}x_{i}+\sum \beta_{ii}x_{i}^{2}+\sum \beta_{ij}x_{i}x_{j}$$

where *Y* represents the response variable; *x_i_* and *x_j_* are the coded independent variables; *β*_0_ is the intercept coefficient; and *β*_i_, *β*_ii_, and *β*_ij_ are the regression coefficients for linear, quadratic, and interaction terms, respectively.

### 2.16. Statistical Analysis

Data were expressed as means. Regression analysis, ANOVA, response surface construction, and contour plots were performed using Statistica 14.0.0.15 (TIBCO Software Inc., Tulsa, OK, USA) at a significance level of 10%. Final model terms were selected by backward elimination, retaining effects with *p* ≤ 0.10 to preserve model hierarchy given the residual degrees of freedom available in the design. Goodness of fit was evaluated by R^2^, adjusted R^2^, *F*-value, CV%, and lack of fit (LoF), with acceptance thresholds of R^2^ adj ≥ 0.70, CV% < 10%, for compositional responses (TSPC and protein content) and CV% < 20% for bioactive and antioxidant capacity responses (GABA, ORAC and FRAP), in accordance with the higher intrinsic biological variability documented for these determinations in germinated matrices, and LoF *p* > 0.05 [19,26]. Differences in reducing sugar content and enzyme activities between ungerminated *kañiwa* and the sample germinated under the optimized conditions were evaluated using Student’s *t*-test. Differences were considered statistically significant at *p* < 0.05. Since the experimental points of the central composite design correspond to a continuous response surface rather than to independent treatment groups, the statistical significance of the effects was assessed through the ANOVA of the regression models rather than through multiple-comparison tests.

### 2.17. AI Use Statement

**Figure 2:**
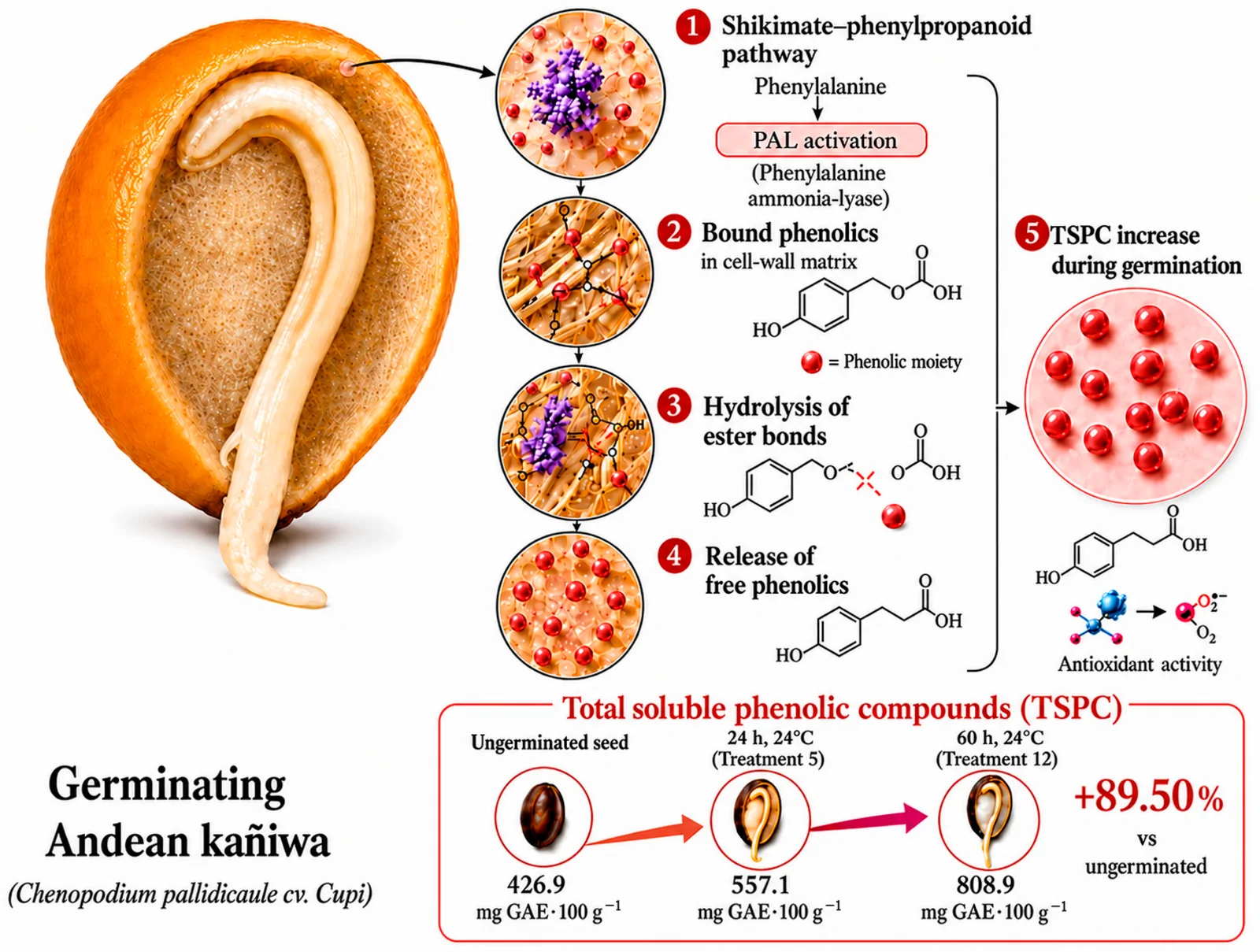
Conceptual representation of the phenylpropanoid pathway associated with TSPC accumulation during *kañiwa* germination, compiled from the cited literature [1,8,10,15,17,45,47,48]. TSPC: total soluble phenolic compounds; PAL: phenylalanine ammonia-lyase; PPO: polyphenol oxidase; GAE: gallic acid equivalent. The enzymatic steps depicted were not measured in the present study; the only variable determined experimentally is TSPC. Image generated with artificial intelligence assistance and reviewed/edited by the authors for illustrative purposes.

**Figure 3:**
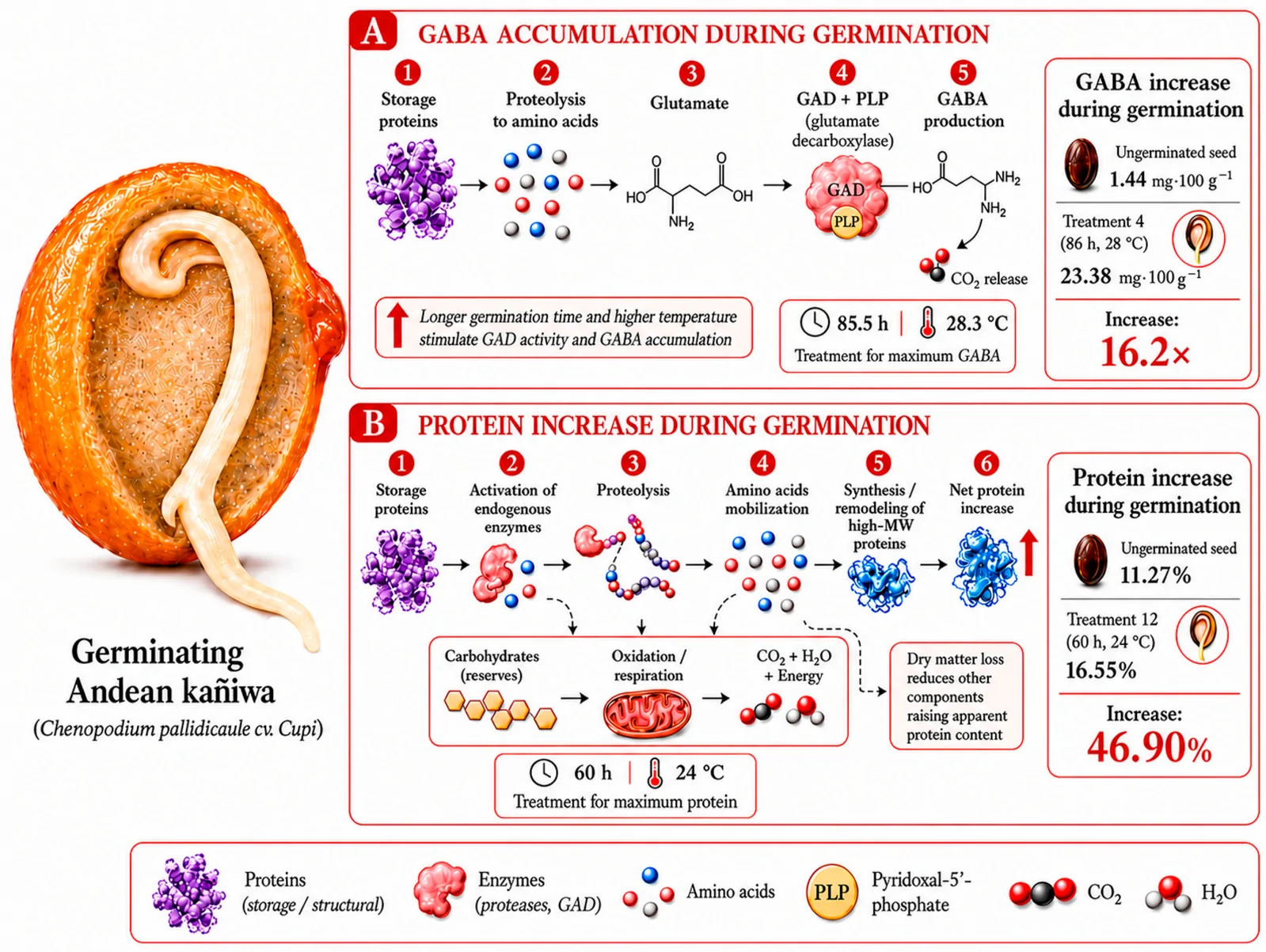
Conceptual representation of the glutamate decarboxylase pathway associated with GABA accumulation and protein increase during *kañiwa* germination, compiled from the cited literature [2,6,7,13,19,21,22]. GABA: *γ*-aminobutyric acid; GAD: glutamate decarboxylase; PLP: pyridoxal 5′-phosphate; MW: molecular weight; ↑: increase; CO_2_: carbon dioxide; H_2_O: water. GAD activity was not measured in the present study; the variables determined experimentally are GABA and protein content. Image generated with artificial intelligence assistance and reviewed/edited by the authors for illustrative purposes.

**Figure 4:**
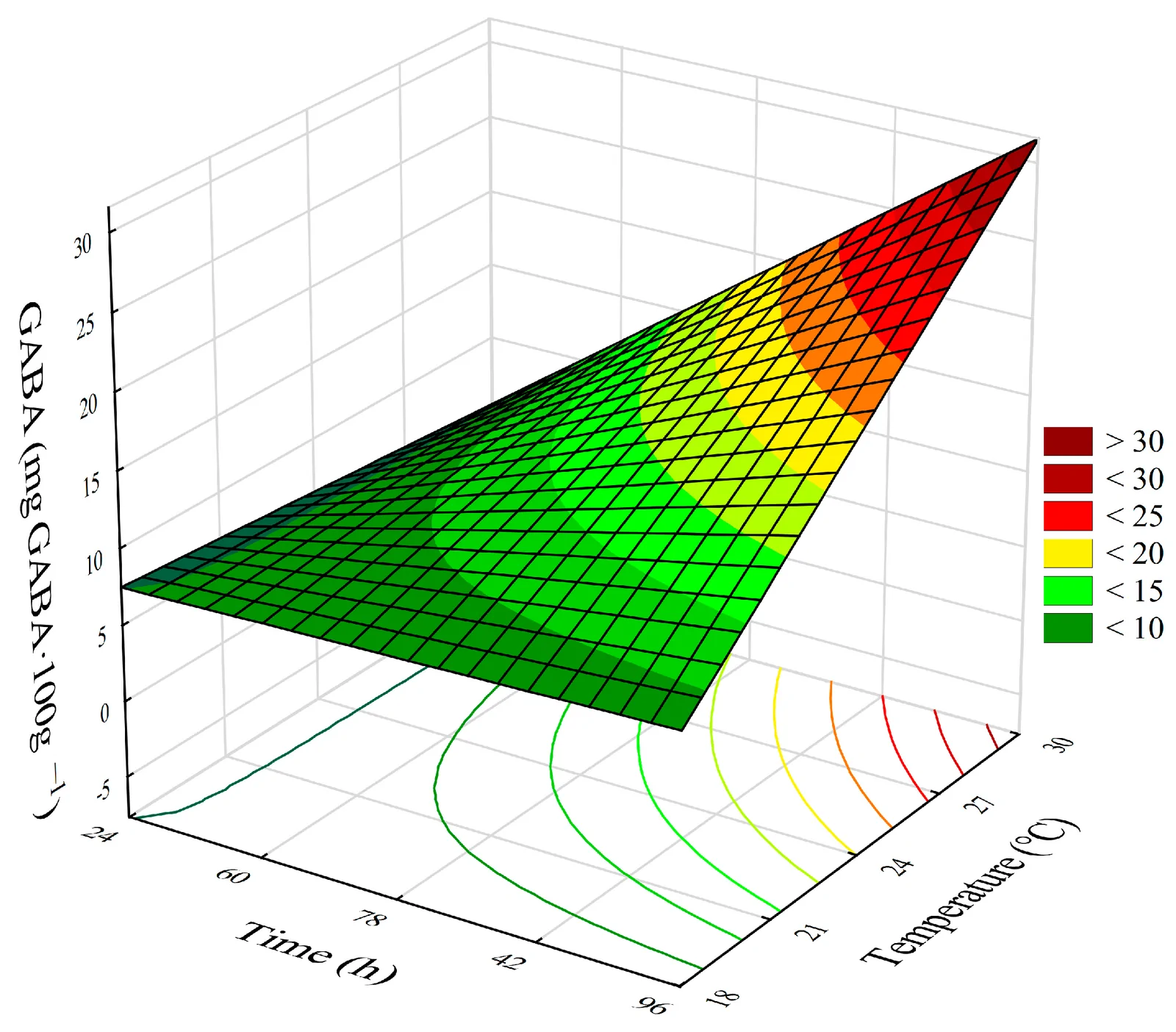
Response surface plot of *γ*-aminobutyric acid (GABA) in mg GABA·100 g^−1^ (d.b.) of germinated *kañiwa* as a function of germination time (h) and temperature (°C).

**Figure 5:**
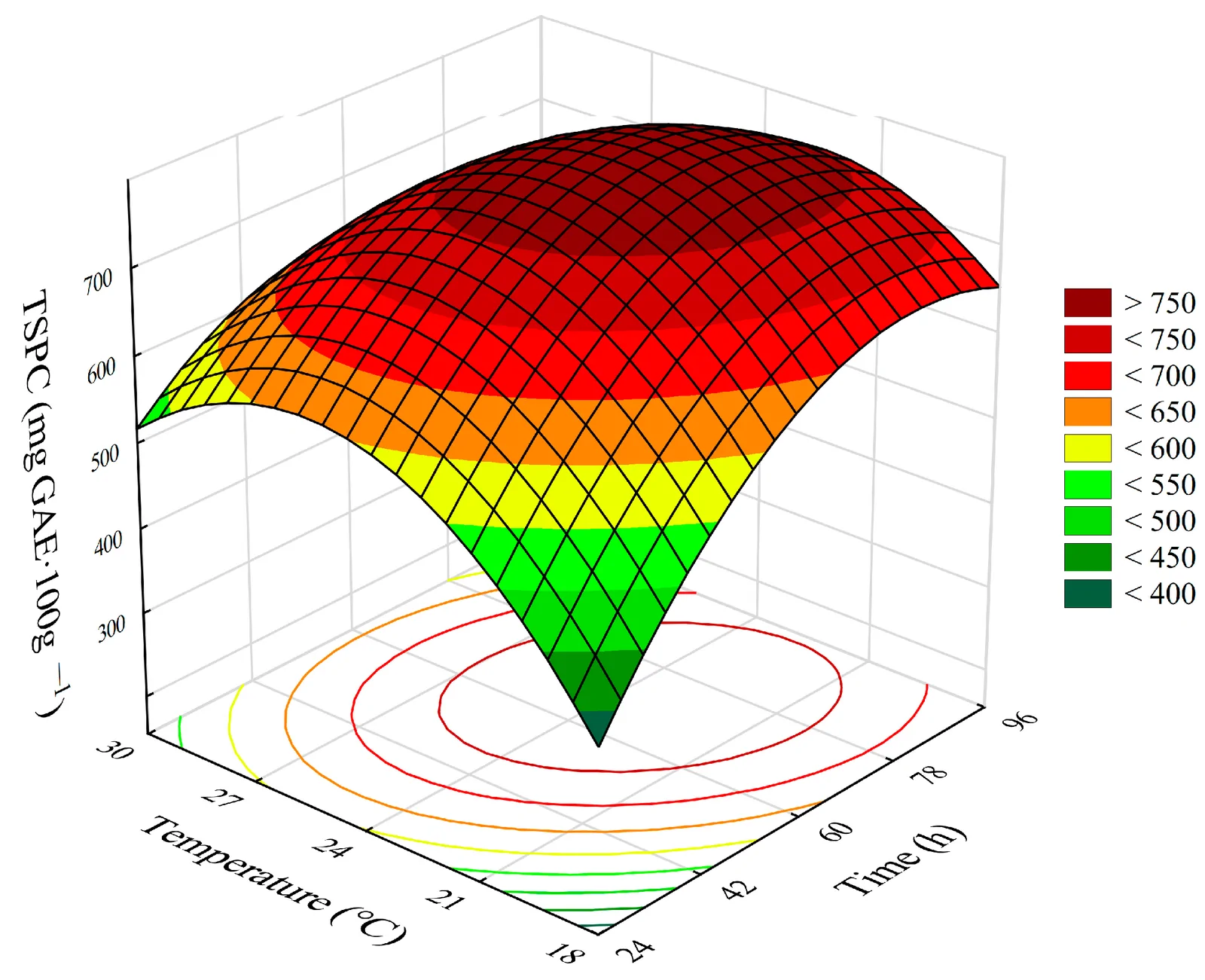
Response surface plot showing the effects of germination time (h) and temperature (°C) on total soluble phenolic compounds (mg GAE·100 g^−1^) in germinated *kañiwa*.

**Figure 6:**
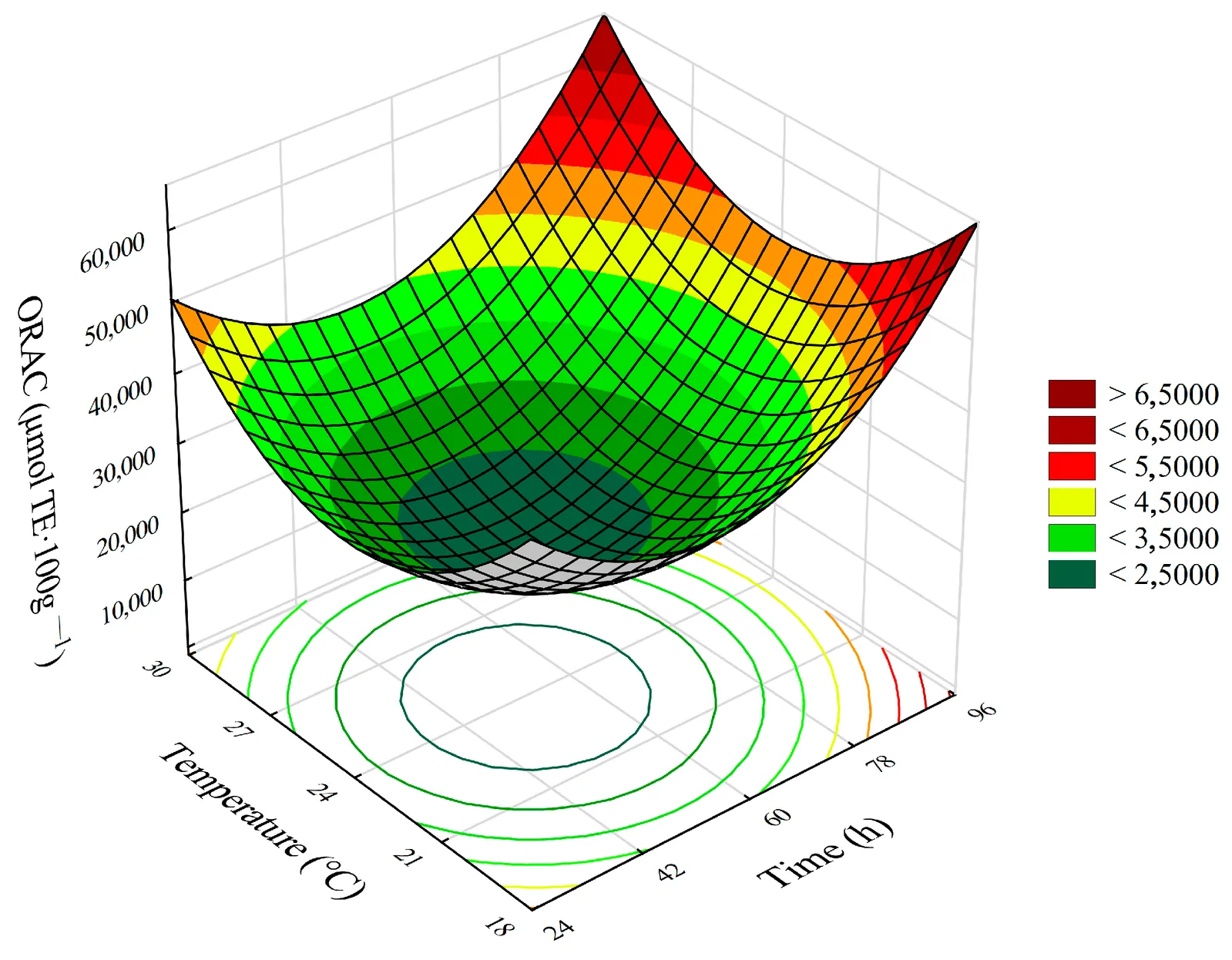
Response surface plot showing the effects of germination time (h) and temperature (°C) on the ORAC antioxidant capacity (µmol TE·100 g^−1^) of germinated *kañiwa*.

**Figure 7:**
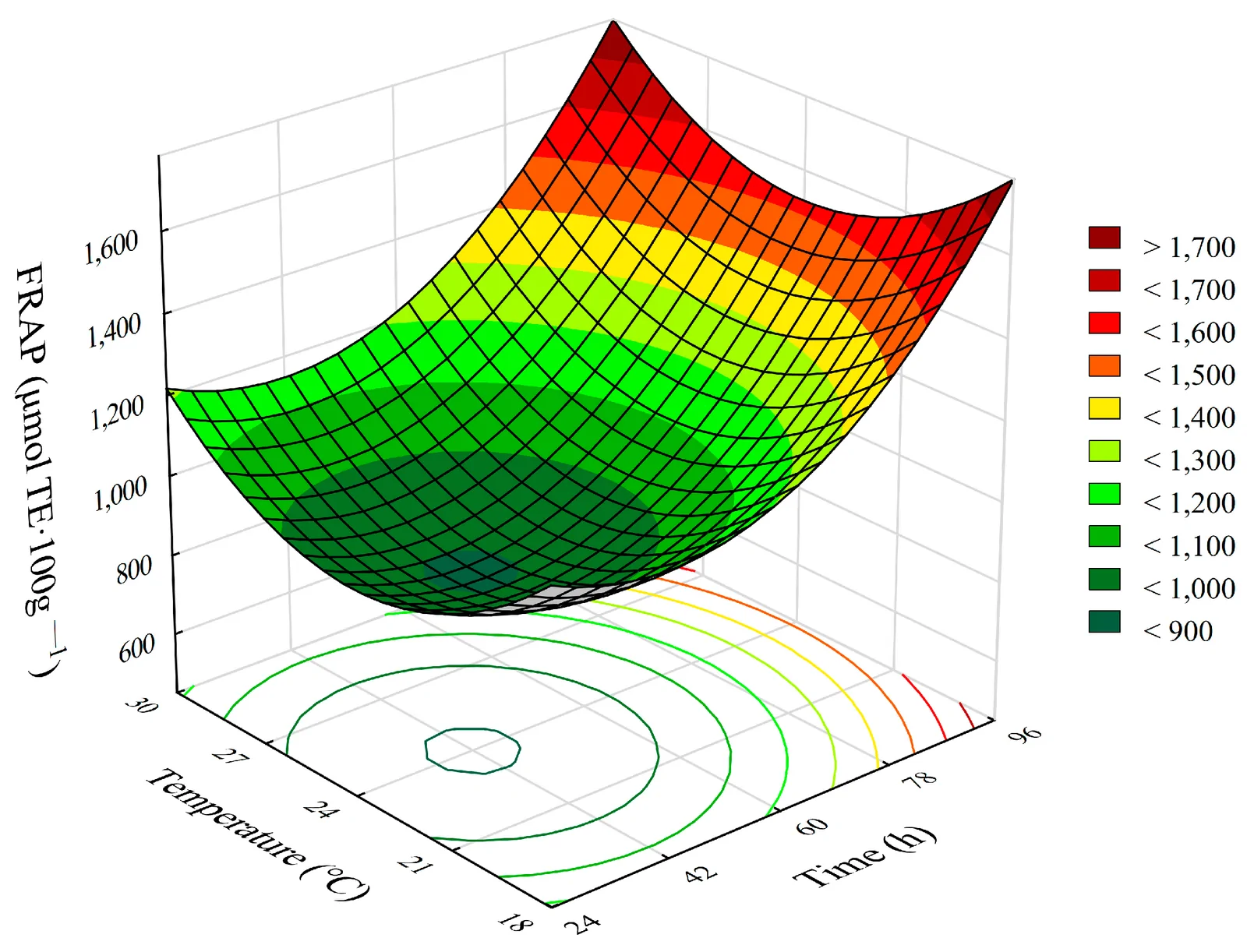
Response surface plot of FRAP (µmol TE·100 g^−1^) of germinated *kañiwa* as a function of germination time (h) and temperature (°C). Axes correspond to the real values of the independent variables.

**Figure 8:**
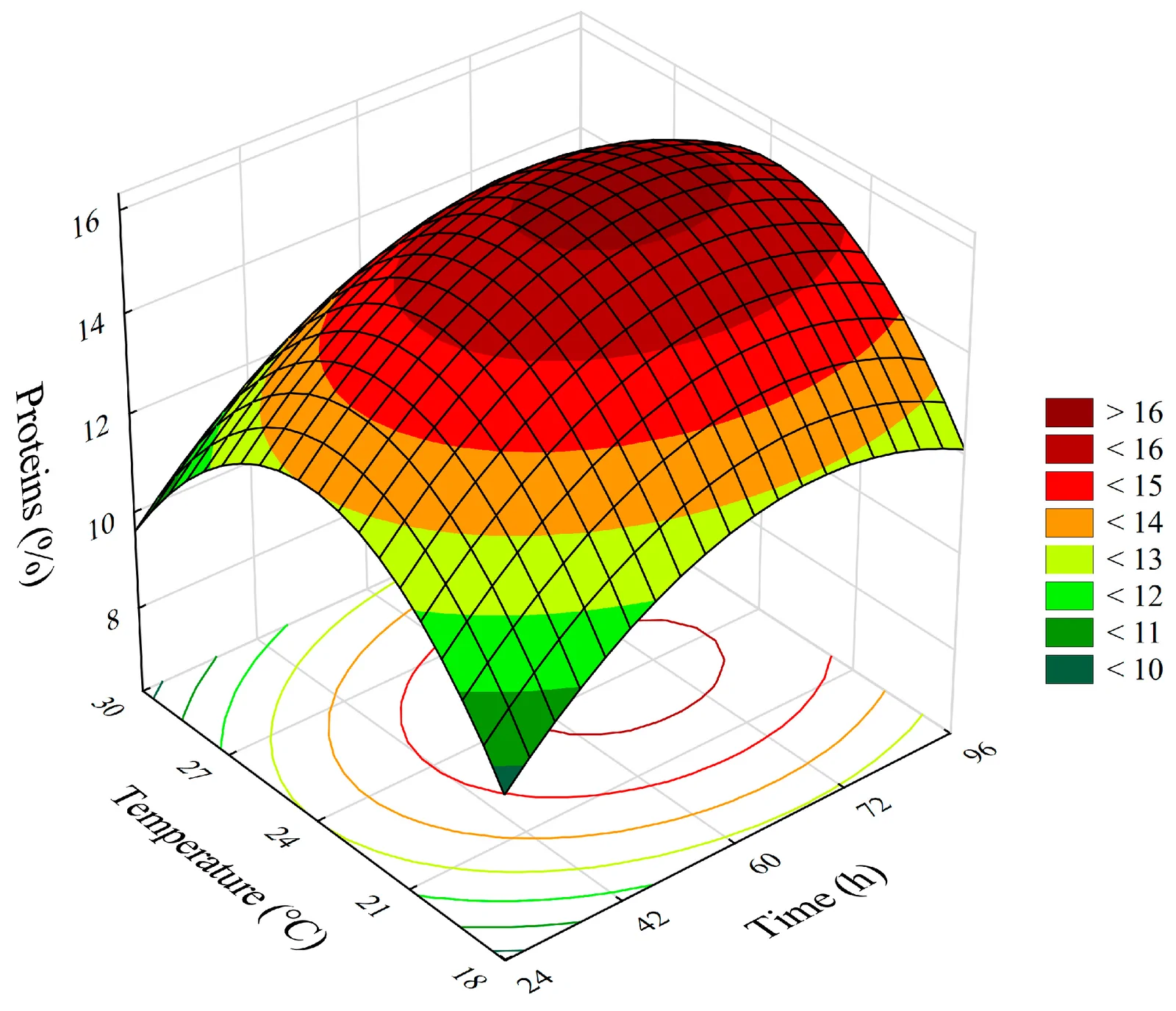
Response surface plot showing the effects of germination time (h) and temperature (°C) on protein content (%) in germinated *kañiwa*.

**Figure 9:**
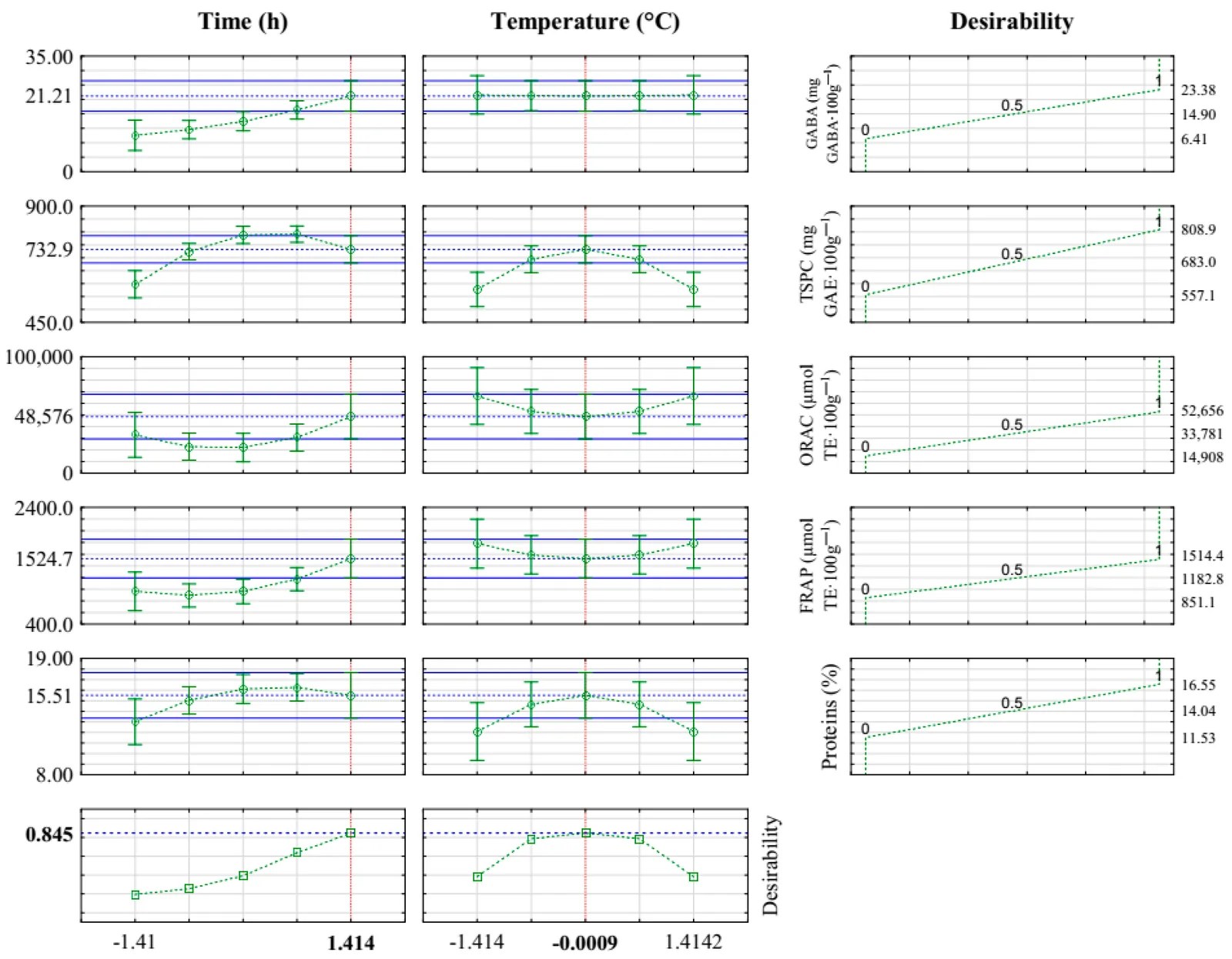
Predicted response profiles and global desirability function for the simultaneous optimization of *kañiwa* germination conditions.

During the preparation of this manuscript, generative artificial intelligence tools were used to assist with translation and linguistic revision of the text, and to generate the conceptual diagrams of Figure 2 and Figure 3 and the graphical abstract, all of which were subsequently reviewed and edited by the authors. The location map (Figure 1) was replaced in this revised version by a map produced by the authors in ArcGIS Desktop 10.5. All response surface contour plots (Figure 4, Figure 5, Figure 6, Figure 7, Figure 8 and Figure 9) were produced in Statistica 14.0.0.15. The conceptualization, methodological design, experimental work, data analysis, interpretation of results and all scientific content were developed and verified entirely by the authors, who take full responsibility for the content of this publication.

## 3. Results and Discussion

### 3.1. Experimental Values of Response Variables in the CCD

The results of the experimental values obtained for GABA, total soluble phenolic compounds (TSPC), antioxidant capacity (ORAC and FRAP), and protein content are presented in Table 3. For comparative purposes, the baseline values of ungerminated *kañiwa*, determined under the same analytical conditions were also included. The results demonstrate that controlled germination markedly influenced all evaluated responses, confirming the strong dependence of *kañiwa* biochemical metabolism on germination conditions.

TSPC values ranged from 557.1 mg GAE·100 g^−1^ (#5: 24 h, 24 °C) to 808.9 mg GAE·100 g^−1^ (#12: 60 h, 24 °C), corresponding to a maximum increase of 89.50% relative to ungerminated seeds. This substantial enhancement is consistent with the activation of the phenylpropanoid pathways described in the literature, although PAL activity was not determined in the present study and the release of bound phenolics during germination, phenomena widely reported for Andean pseudocereals [17,45].

ORAC exhibited the greatest relative variation among the antioxidant responses, ranging from 14,908.5 µmol TE·100 g^−1^ (#12) to 52,655.7 µmol TE·100 g^−1^, with the highest value observed in trial #6 (96 h, 24 °C). This represents a 267.70% increase compared with ungerminated *kañiwa*. Similarly, FRAP values ranged from 851.1 µmol TE·100 g^−1^ to 1514.4 µmol TE·100 g^−1^, with a maximum response obtained in trial #2 (85.5 h, 19.7 °C), corresponding to a 164.34% increase over the control. The simultaneous increase in ORAC and FRAP values **is** compatible with an enhancement of both hydrogen atom transfer and single-electron transfer antioxidant mechanisms, thereby broadening the overall antioxidant potential of *kañiwa*. These observed metrics are highly consistent with established baselines for Andean pseudocereals cultivated under controlled conditions [13,15,45].

Among all responses evaluated, GABA showed the greatest relative increase, rising from 1.44 mg·100 g^−1^ in ungerminated seeds to 23.38 mg·100 g^−1^ in trial #4 (85.5 h, 28.3 °C), representing a 16.20-fold increase. This behavior is consistent with the activation of glutamate decarboxylase (GAD) during controlled germination, whose activity is strongly influenced by germination time and temperature [7,21,46]. The values observed in germinated *kañiwa* are the first documented for this species under controlled germination conditions [2,13]. Notably, these results constitute the first quantitative report of GABA accumulation in *kañiwa* under controlled germination conditions and provide the first experimental basis for optimizing this bioactive compound in the species.

Figure 2 and Figure 3 illustrate the proposed biochemical mechanisms underlying the accumulation of phenolic compounds and GABA, respectively, during *kañiwa* germination, providing a mechanistic framework for interpreting the responses observed throughout the experimental design.

Protein content increased from 11.27% in ungerminated seeds to a maximum of 16.55% in trial #12 (60 h, 24 °C), representing a 46.90% increase. This increase can be attributed to the combined effects of reserve protein mobilization, de novo synthesis of enzymatic proteins, and the reduction in carbohydrate reserves through respiration, which concentrates the protein fraction on a dry-weight basis [13,15,46]. The observed protein enrichment further reinforces the potential of germinated *kañiwa* as a nutrient-dense ingredient for functional food applications.

The four central points (#9–12; 60 h, 24 °C) exhibited low experimental variability, with TSPC values between 760.9 and 808.9 mg GAE·100 g^−1^ and a standard deviation of 20.93 mg GAE·100 g^−1^ (CV = 2.65%), confirming the repeatability of the experimental system and the stability of the central region of the design. These results support the reliability of the experimental data and the subsequent response surface modeling approach. [14,29].

### 3.2. Fitting Second-Order Polynomial Models

The five second-order polynomial models demonstrated satisfactory goodness-of-fit, with coefficients of determination (R^2^) ranging from 0.784 for ORAC and 0.912 for GABA, while adjusted coefficients of determination (R^2^ adj) varied between 0.703 and 0.880, all exceeding the minimum acceptance criterion of 0.700 established for predictive response surface models [14,23,26]. Analysis of variance (ANOVA) revealed that all regression models were highly significant, with *p*-values ranging from <0.001 to 0.003 (Table 4), indicating that the fitted equations successfully explained a substantial proportion of the experimental variability. The highest statistical significance was observed for the GABA model (*F* = 27.77; *p* < 0.001), followed by protein content (*F* = 16.04; *p* = 0.001), TSPC (*F* = 12.49; *p* = 0.003), FRAP (*F* = 11.58; *p* = 0.003), and ORAC (*F* = 11.19; *p* = 0.003). Furthermore, the lack-of-fit test was non-significant (*p* > 0.050) for all responses, confirming that the residual variation was adequately explained by experimental error rather than systematic deviations from the fitted models. The models met the criteria of significant regression and non-significant lack of fit in all five cases. R^2^adj exceeded the threshold of 0.700 for all responses, ranging from 0.703 (ORAC) to 0.880 (GABA). CV% remained below 10.00% for TSPC (5.52%) and protein content (5.97%); higher values were observed for GABA (15.77%), ORAC (18.30%) and FRAP (10.90%), which did not meet the conventional 10.00% threshold but remained within the 20.00% limit adopted for bioactive and antioxidant capacity responses, reflecting the greater intrinsic biological variability of these determinations in germinated matrices [14,23]. The statement that all models simultaneously satisfied all criteria is not accurate and has been corrected accordingly; the models are presented as adequate for describing response trends and locating the region of the optimum within the experimental domain [26,29,30,45].

The coefficient variation (CV) was considered satisfactory for TSPC (5.52%) and protein content (5.97%), both remaining below the conventional threshold of 10.00% commonly adopted for response surface studies. Higher CV values were observed for ORAC (18.30%), GABA (15.77%), and FRAP (10.90%), reflecting the greater biological sensitivity of these responses to variations in germination time and temperature. Similar levels of variability have been reported for antioxidant and bioactive responses in germinated pseudocereals, including quinoa and amaranth, evaluated under comparable RSM approaches [14,23]. These results collectively confirm that the fitted models provide an adequate description of the experimental domain and are suitable for identifying the region of the optimum within the evaluated range [9,26,29].

Analysis of the regression coefficients revealed distinct response patterns among the evaluated variables. Germination time exerted a significant positive linear effect on TSPC, ORAC, FRAP, and GABA (*p* < 0.050), demonstrating its central role in promoting the accumulation of bioactive compounds during sprouting. In contrast, the quadratic effect of time was significant for TSPC and ORAC, indicating the presence of optimal germination periods beyond which the responses tended to stabilize or decline. Temperature showed a significant linear effect only on GABA accumulation (*p* = 0.015), whereas significant quadratic effects were detected for TSPC, ORAC, and protein content, suggesting a non-linear modulation of metabolic and enzymatic activities associated with germination. The interaction between time and temperature was significant for TSPC (*p* = 0.039) and approached significance for GABA (*p* = 0.058), indicating that the effect of one factor depended partially on the level of the other. These results demonstrate that the accumulation of bioactive compounds in *kañiwa* is governed by a complex interplay between germination duration and temperature, reinforcing the importance of multivariate optimization strategies to identify the most favorable processing conditions.

The regression coefficients of the final second-order polynomial models for the five response variables are presented in Table 5 and constitute the basis for the construction and interpretation of the response surfaces discussed in the following sections.

The signs of the quadratic coefficients were reflected directly in the morphology of the response surfaces. Negative quadratic terms (*β*_11_ and *β*_22_) for TSPC and protein content produced concave surfaces with clear interior optima, generating the hill-shaped profiles observed in the contour plots. Conversely, positive quadratic coefficients for ORAC and FRAP resulted in convex surfaces characterized by valley-shaped regions and progressive increases toward the boundaries of the experimental domain. For GABA, no significant quadratic effects were detected, indicating that the response was governed primarily by linear effects of germination time and temperature. Consequently, the response surface exhibited a monotonic gradient rather than a stationary point, suggesting that higher GABA accumulation may still be achieved beyond part of the evaluated experimental range [26].

### 3.3. Effect of Germination Temperature and Time on GABA Content

The fitted second-order polynomial model for GABA included three significant effects (Table 4): the linear effect of germination time (*F* = 42.28, *p =* 0.007), the linear effect of germination temperature (*F* = 24.97, *p =* 0.015), and the time × temperature interaction (*F* = 9.01, *p =* 0.058). The quadratic effects of both factors were non-significant and therefore excluded from the final model. This behavior differed markedly from that observed for the other response variables and indicates that GABA accumulation in germinated *kañiwa* was governed primarily by linear and interaction effects, with no evidence of a stationary point or interior optimum within the experimental domain. The dominant contribution was associated with germination time (|t| = 6.50), followed by temperature (|t| = 5.00), demonstrating that both independent variables exerted a continuous positive effect on GABA biosynthesis throughout the evaluated range (24–96 h; 18–30 °C) [7,23,45,46]. The positive interaction term (*p =* 0.058) further suggests that the stimulatory effect of germination time became more pronounced at higher temperatures, indicating a synergistic relationship between both process variables [7,21]. The final fitted polynomial model for GABA, expressed in coded variables, is shown in Equation (4). The absence of quadratic terms in this model is consistent with the monotonically increasing gradient observed in the response surface (Figure 4).

(4) $$GABA=13.24+4.81x_{1}+3.70x_{2}+3.14x_{1}x_{2}$$

where GABA is the content of *γ*-aminobutyric acid expressed as mg of GABA·100 g^−1^ (d.b.); x_1_ and x_2_ represent the coded level for germination time and temperature, respectively.

The absence of quadratic coefficients is consistent with the morphology of the response surface, which exhibited a continuously increasing gradient rather than the hill-shaped profiles observed for TSPC and protein content.

The contour plot and response surface shown in Figure 4 reveal a progressive increase in GABA concentration from the lower region of the experimental domain (short germination times and low temperatures; <6 mg·100 g^−1^) toward the upper region characterized by prolonged germination periods and elevated temperatures. Predicted values exceeded 40 mg·100 g^−1^ in the upper-right portion of the response surface, and the highest-response region appeared to extend beyond the boundaries of the evaluated design. This behavior contrasts with that observed for TSPC, which presented a well-defined interior optimum, and suggests that the conditions required to maximize GABA accumulation may not have been fully reached within the experimental range investigated. Such a response is consistent with the continuous activation of glutamate decarboxylase (GAD) and the sustained conversion of glutamic acid into GABA under favorable germination conditions, reinforcing the strong dependence of GABA biosynthesis on both germination duration and temperature [7,14,23].

The GABA concentrations observed in germinated *kañiwa* (6.41–23.38 mg GABA·100 g^−1^, d.b.) represent the first quantitative report for this species under controlled germination conditions and constitute the first attempt to model and optimize GABA accumulation through Response Surface Methodology [2,13]. The relatively high glutamic acid content naturally present in *kañiwa* (16.91 g·100 g^−1^ protein) [2,3], which serves as the direct substrate for glutamate decarboxylase (GAD), provides a favorable biochemical basis for GABA biosynthesis during germination. Although the concentrations obtained were lower than those reported for amaranth germinated at 26 °C for 63 h (75.70 mg GABA·100 g^−1^; 29.00-fold increase) [23] and purple maize under similar conditions (74.40 mg GABA·100 g^−1^) [14,20], they confirm the capacity of *kañiwa* to accumulate physiologically relevant amounts of this bioactive metabolite through controlled germination. Moreover, the observed values are within the range reported for several germinated cereals and pseudocereals subjected to process optimization strategies. For example, optimization of soaking and germination conditions in foxtail millet resulted in 26.35 mg GABA·100 g^−1^ with a highly predictive model (R^2^ = 99.69%) [21], highlighting the strong responsiveness of GABA biosynthesis to process variables [2,13,46].

The accumulation of GABA during germination has been attributed in the literature to the activation of GAD; this enzyme was not quantified in the present study, so the following interpretation constitutes a hypothesis supported by previous work rather than an experimentally demonstrated mechanism, a pyridoxal 5′-phosphate (PLP)-dependent enzyme that catalyzes the irreversible conversion of *L*-glutamic acid into GABA and CO_2_ [6,7,46]. According to the literature, germination has been associated with the activation of GAD as part of the seed metabolic reprogramming process, with reported activity generally increasing with both germination time and temperature and reaching maximum levels near 30 °C in several cereals and pseudocereals [7,14,21]; this enzyme was not measured in the present study. This mechanism is consistent with the significant positive linear effects of time and temperature observed in the present study, which indicate a continuous stimulation of GABA biosynthesis throughout the evaluated experimental domain. The absence of significant quadratic effects further suggests that the enzymatic system has not yet reached a saturation or inhibition phase under the tested germination conditions. Prolonged germination has also been reported to enhance the action of endogenous proteases, which hydrolyze storage proteins and release free amino acids, including glutamic acid, the direct substrate for GABA synthesis [7,19,21]. The significant positive interaction between germination time and temperature indicates that these factors acted synergistically to increase both substrate availability and enzymatic activity. Consequently, higher temperatures may accelerate metabolic reactions associated with protein mobilization and amino acid turnover, while longer germination periods provide sufficient time for the progressive accumulation of GABA. The combined action of these processes provides a mechanistic explanation for the monotonic increase in GABA concentration observed in the response surface and supports the interpretation that the maximum biosynthetic potential of GABA was not reached within the experimental range investigated [2,7,46].

### 3.4. Effect of Germination Temperature and Time on Total Soluble Phenolic Compounds (TSPC)

The fitted second-order polynomial model for TSPC included four significant effects (Table 4): linear germination time (*F* = 41.72, *p* = 0.008), quadratic time (*F* = 55.49, *p* = 0.005), quadratic temperature (*F* = 87.78, *p* = 0.003), and the time × temperature interaction (*F* = 12.30, *p* = 0.039). The linear effect of temperature was not significant and was therefore excluded from the final model. Pareto analysis revealed that the dominant contribution was associated with the quadratic temperature effect (|t| = −9.37), followed by quadratic time (|t| = −7.45) and linear time (|t| = 6.46). These results indicate that TSPC accumulation was governed predominantly by non-linear responses to both germination variables, resulting in a well-defined optimum within the experimental domain. Furthermore, the significant interaction term (|t| = −3.51) demonstrates that the effect of germination time depended on temperature, confirming a non-additive relationship between the two factors [14,23,45]. The final polynomial model for TSPC, expressed in coded variables, is presented in Equation (5).

(5) $$TSPC=788.6+47.80x_{1}-61.63x_{1}^{2}-77.50x_{2}^{2}-36.70x_{1}x_{2}$$

where TSPC is the content of total soluble phenolic compounds expressed as mg of gallic acid equivalent·100 g^−1^ (d.b.); x_1_ and x_2_ represent the coded level for germination time and temperature, respectively.

The response surface shown in Figure 5 exhibited a characteristic hill-shaped morphology, with a clearly defined interior maximum located in the central region of experimental space. The highest predicted TSPC values (>700 mg GAE·100 g^−1^) were concentrated between approximately 40–80 h of germination and temperatures ranging from 20 to 28 °C, defining a relatively broad optimal region. Beyond this range, TSPC progressively declined, particularly at extended germination periods (96 h) and at temperatures approaching the experimental limits. This behavior is consistent with the negative quadratic coefficients of time (β_11_ = −61.63) and temperature (β_22_ = −77.51), confirming that phenolic accumulation was maximized only under intermediate germination conditions. Unlike GABA, which exhibited a continuously increasing response, TSPC presented a stationary point within the evaluated domain, indicating that phenolic biosynthesis and release were eventually counterbalanced by degradation or utilization processes [15,45].

The TSPC values obtained in the present study (557.1–808.9 mg GAE·100 g^−1^) substantially exceeded the baseline value of ungerminated *kañiwa* (426.9 mg GAE·100 g^−1^), corresponding to a maximum increase of 89.50%. These concentrations are among the highest reported for germinated Andean pseudocereals when expressed on a dry-matter basis. Previous studies reported TSPC values of 452.0 mg GAE·100 g^−1^ in quinoa germinated at 25 °C for 72 h [16,46], and 385.4 mg GAE·100 g^−1^ in *kiwicha* germinated at 26 °C for 63 h [23], while in amaranth germinated at 30 °C for 78 h, an 829.0% increase in total phenolics was documented [15]. Moreover, Repo-Carrasco-Valencia et al. [1] reported only a 17% increase in extractable phenolics in *kañiwa* germinated at 20 °C for 72 h. The substantially greater increase observed in the present study highlights the effectiveness of response surface methodology for identifying germination conditions that maximize the bioactive potential of *kañiwa*, while also reinforcing the importance of using optimized extraction procedures for phenolic recovery [10,45].

The increase in TSPC during germination can be explained by two complementary biochemical mechanisms. The first involves the activation of phenylalanine ammonia-lyase (PAL), the key regulatory enzyme of the shikimate–phenylpropanoid pathway, which catalyzes the conversion of *L*-phenylalanine into *trans*-cinnamic acid and initiates the biosynthesis of a wide range of phenolic metabolites [6,17,25,46]. The second mechanism involves the hydrolysis of ester and ether linkages between phenolic compounds and cell-wall polysaccharides, mediated by endogenous hydrolytic enzymes that release previously bound phenolics into extractable forms [8,13,17]. The existence of a temporal maximum in TSPC, reflected by the negative quadratic coefficient for germination time, is consistent with observations reported for *Chenopodium album* [45] and quinoa [14]. This behavior can be explained by the dynamic balance between phenolic biosynthesis, release, and degradation throughout germination. During the early and intermediate stages, PAL activation and the release of bound phenolics reported in the literature are thought to predominate, which would be consistent with the progressive increase in TSPC observed. However, as germination advances, oxidative and catabolic processes become increasingly important. In particular, polyphenol oxidase (PPO) activity may accelerate the oxidation of phenolic compounds, causing the rate of degradation to exceed the rate of synthesis and release, ultimately resulting in a decline in extractable phenolic content [8,10,17]. This biochemical transition provides a mechanistic explanation for the hill-shaped response surface observed in Figure 5 and for the existence of an optimal germination period to maximize phenolic accumulation in *kañiwa*.

### 3.5. Effect of Germination Temperature and Time on Antioxidant Capacity Assessed by ORAC

The fitted second-order polynomial model for ORAC included three significant effects (Table 4): linear time (*F* = 4.16, *p* = 0.037), quadratic time (*F* = 9.58, *p* = 0.005), and quadratic temperature (*F* = 8.50, *p* = 0.007). The dominant contribution was associated with the quadratic effect of time (|t| = 3.80), followed by quadratic temperature (|t| = 3.58) and linear time (|t| = 2.51), indicating that ORAC was influenced predominantly by non-linear responses to both germination variables, with an additional positive contribution from germination duration. Among the evaluated responses, ORAC exhibited the lowest predictive performance (R^2^ adj = 0.703; Table 4), reflecting the greater biological variability commonly reported for antioxidant capacity measurements in germinated pseudocereals [14,23]. The high CV for ORAC (18.30%) does not reflect analytical imprecision but rather the intrinsic biological variability of the germination process: the four independent germinations performed at the central point (60 h; 24 °C) yielded ORAC values between 14,908.5 and 32,687.2 µmol TE·100 g^−1^, a coefficient of variation of 34.91%, whereas the intra-assay relative standard deviation of the triplicate determinations ranged from 0.90% to 7.95% (Table 3). The non-significant lack of fit (*F* = 0.46; *p* = 0.790) confirms that this variability is not attributable to an inadequate model form but is inherent to the response. The final fitted polynomial model for ORAC, expressed in coded variables, is shown in Equation (6).

(6) $$ORAC=21,978+5534.2x_{1}+9387.3x_{1}^{2}+8841.8x_{2}^{2}$$

where ORAC (Oxygen Radical Absorbance Capacity) is the antioxidant capacity expressed as µmol of trolox equivalent·100 g^−1^ (d.b.); x_1_ and x_2_ represent the coded level for germination time and temperature, respectively.

The response surface (Figure 6) presented a characteristic basin-shaped (valley-shaped) morphology, with the lowest predicted ORAC values located in the central region of the experimental domain and progressively higher values toward longer germination periods and temperatures farther from the central point. This pattern is consistent with the positive quadratic coefficients identified for both variables. The highest experimental ORAC value (52,655.7 µmol TE·100 g^−1^) was obtained in #6 (96 h, 24 °C), corresponding to a 267.70% increase relative to ungerminated *kañiwa*, whereas the lowest value among the central-point replicates was 14,908.5 µmol TE·100 g^−1^ (#12). These results indicate that the central region of the experimental design was suboptimal for maximizing peroxyl radical scavenging capacity and that prolonged germination played a particularly important role in enhancing antioxidant activity [1,23].

The ORAC values obtained in germinated *kañiwa* (14,908.5–52,655.7 µmol TE·100 g^−1^) exceeded those reported for several Andean pseudocereals evaluated under non-optimized germination conditions. In amaranth optimized by RSM (26 °C, 63 h), ORAC reached 10,090.2 µmol TE·100 g^−1^ [23], whereas germinated quinoa exhibited an increase of approximately 130.00% relative to ungerminated seeds [14]. Similarly, amaranth germinated under optimized conditions showed a 300.00% increase in ORAC compared with the ungerminated grain [15]. The elevated ORAC values observed in the present study are consistent with the naturally high abundance of phenolic compounds and flavonoids reported for *kañiwa*, particularly derivatives of quercetin and isorhamnetin [1,3]. Furthermore, the simultaneous increase in TSPC and ORAC supports the hypothesis that germination enhanced the pool of compounds capable of scavenging peroxyl radicals.

The biochemical mechanism underlying the ORAC increase during germination is primarily linked to the accumulation of compounds capable of interrupting lipid peroxidation chains mediated by peroxyl radicals (ROO•), including free phenolics synthesized via the PAL/shikimate–phenylpropanoid pathway, flavonoids released by enzymatic hydrolysis, and ascorbic acid whose biosynthesis is activated during germination [13,17,23]. The positive correlations between TSPC and ORAC previously reported for germinated *kiwicha* (r = 0.894, *p* < 0.050) [23] and quinoa (r = 0.760) [14] support the important contribution of phenolic compounds to the peroxyl radical scavenging capacity of germinated pseudocereals [8,22,46]. However, the contrasting response surface morphologies observed in the present study, a hill-shaped profile for TSPC and a valley-shaped profile for ORAC, suggest that antioxidant capacity was not determined solely by the total concentration of phenolic compounds. Instead, ORAC responses were likely influenced by qualitative changes in the phenolic profile, including the accumulation of compounds with higher antioxidant activity, as well as by the synthesis of other antioxidant metabolites during germination. The increase in ORAC observed at extended germination periods may therefore reflect not only greater activation of the shikimate–phenylpropanoid pathway but also the progressive formation of secondary antioxidants that contribute to peroxyl radical scavenging capacity [1,10,17].

This interpretation highlights that germination affects both the quantity and the antioxidant effectiveness of bioactive compounds in *kañiwa*. The divergence between the TSPC and ORAC surfaces reflects a well-documented dissociation between total phenolic content and antioxidant capacity: these assays quantify chemically distinct properties—total reducing capacity, peroxyl-radical scavenging through hydrogen-atom transfer, and ferric-reducing power through single-electron transfer, respectively—so that the ranking of a set of samples depends on the assay employed [49,50]. This behavior has been reported specifically for *C. pallidicaule*, in which antioxidant capacity at 96 h of germination did not match extractable flavonoid content, indicating that antioxidant compounds other than the extractable phenolic fraction are induced during germination [1].

### 3.6. Effect of Germination Temperature and Time on Antioxidant Capacity Assessed by FRAP

The fitted second-order polynomial model for FRAP included three effects (Table 4): linear time (*F* = 18.27, *p* = 0.024), quadratic time (*F* = 7.29, *p* = 0.074), and quadratic temperature (*F* = 6.03, *p* = 0.091). Although the quadratic terms showed marginal significance, their *p*-values were within the 0.070–0.090 range and were retained in the final model due to their contribution to describing the curvature of the response surface within the 12-trial experimental design [23,29]. The dominant effect was the positive linear effect of germination time (|t| = 4.28), indicating that prolonged germination was the most consistent factor promoting Fe^3+^ reducing power in *kañiwa* [8,17,51]. The final fitted polynomial model for FRAP, expressed in coded variables, is shown in Equation (7).

(7) $$FRAP=962.5+198.9x_{1}+140.5x_{1}^{2}+127.7x_{2}^{2}$$

where FRAP (Ferric Reducing Antioxidant Power) is the antioxidant capacity expressed as µmol of Trolox equivalent·100 g^−1^ (d.b.); x_1_ and x_2_ represent the coded level for germination time and temperature, respectively.

The response surface for FRAP (Figure 7) exhibited a valley-shaped morphology, with the lowest predicted values located near the central region of the experimental design and higher values projected toward the boundaries of the experimental domain. This behavior is consistent with the positive quadratic coefficients for germination time (β_11_ = 140.5) and temperature (β_22_ = 127.7). The highest experimental FRAP value was observed in #2 (85.5 h, 19.7 °C), reaching 1514.4 µmol TE·100 g^−1^, which represented a 164.00% increase compared with ungerminated *kañiwa*. Although the response surface suggests higher reducing capacity under more extreme combinations of time and temperature, the strongest experimental response was associated with prolonged germination at a relatively low temperature. This indicates that FRAP enhancement in germinated *kañiwa* may depend not only on the total amount of soluble phenolics but also on the type and redox efficiency of the antioxidant compounds formed during germination [1,8,17].

The FRAP values obtained in germinated *kañiwa* (851.1–1514.4 µmol TE·100 g^−1^) indicate a substantial increase in electron-donating capacity compared with ungerminated seeds. Similar improvements in reducing power have been reported in other germinated grains and pseudocereals. In quinoa, FRAP increased significantly during germination and reached maximum levels after prolonged sprouting periods [8]. Likewise, ultrasound-assisted germination of quinoa increased FRAP by up to 48.04% over the control [52], while soybean germinated for three days at 30 °C showed increases in FRAP activity ranging from 28.62% to 85.91% relative to raw seeds [51]. These comparisons reinforce the effectiveness of germination as a strategy to enhance reducing power, although direct comparisons among studies should account for differences in species, extraction procedures, analytical conditions, and units of expression.

The biochemical basis for the increase in FRAP is the accumulation of electron-donating compounds that reduce the Fe^3+^–TPTZ complex to Fe^2+^–TPTZ under acidic conditions. According to the literature, these compounds include phenolics synthesized through PAL activation, flavonoids released from bound forms, ascorbic acid, peptides, and other low-molecular-weight reducing compounds generated during seed metabolic reprogramming [1,8,13]. Positive associations between FRAP and phenolic compounds have been reported in germinated soybean (r = 0.431, *p* < 0.050) [51] and quinoa [8], supporting the contribution of phenolics to reducing power. The similarity between the FRAP and ORAC response surfaces, both showing valley-shaped patterns, and their divergence from the hill-shaped TSPC surface suggest that antioxidant capacity in germinated *kañiwa* was not exclusively determined by total soluble phenolic concentration, which is consistent with the well-established observation that global spectrophotometric assays quantify chemically distinct mechanisms that do not necessarily track one another [49,50]. Qualitative changes in the antioxidant profile, including the formation of compounds with greater electron-donating capacity, likely contributed to the FRAP response during germination [1,10,17,46].

### 3.7. Effect of Germination Temperature and Time on Protein Content

The fitted second-order polynomial model for protein content included three effects (Table 4): quadratic temperature (*F* = 25.48, *p* = 0.015), linear time (*F* = 8.49, *p* = 0.063), and quadratic time (*F* = 7.43, *p* = 0.072). The greatest impact was promoted by the quadratic effect of temperature, which exhibited a negative coefficient (|t| = −5.05), indicating that germination temperature exerted the strongest influence on protein content. This response suggests the existence of an optimal thermal window around the center of the experimental domain, where protein concentration reached its highest values. The effects of time (linear and quadratic) showed marginal significance (*p* ≈ 0.063–0.072), indicating that germination duration also contributed to protein enrichment, although to a lesser extent than temperature [10,13,15]. The final fitted polynomial model for protein content, expressed in coded variables, is shown in Equation (8).

(8) $$Protein\;content=16.11+0.880x_{1}-0.920x_{1}^{2}-1.71x_{2}^{2}$$

where protein content is expressed as a percentage (d.b.) and x_1_ and x_2_ represent the coded level for germination time and temperature, respectively.

The response surface presented an elongated hill-shaped morphology, with the optimum region located at temperatures close to 24 °C and germination times between approximately 50 and 86 h (Figure 8). The highest predicted protein values (>16%) were concentrated in this central region, whereas lower values were observed at the temperature extremes regardless of germination time. This behavior is consistent with the dominant negative quadratic temperature coefficient and indicates that moderate germination temperatures provide the most favorable balance between metabolic activation and reserve mobilization. At higher temperatures, the acceleration of respiration and proteolytic processes may reduce the net protein fraction, whereas lower temperatures can limit enzyme activation and metabolic reorganization during germination [10,13,46].

The protein contents observed in germinated *kañiwa* (12.51–16.55%) exceeded the baseline value of ungerminated seeds (11.27%), with a maximum increase of 46.90% recorded at the central points of the design (#9–12; 60 h, 24 °C). These values are consistent with the protein range reported for *kañiwa* in the literature (13.92–19.56%) [53] and with the increases documented for other germinated pseudocereals. In quinoa, protein content increased between 1.00% and 26.00%, with improvements attributed to reserve mobilization and the synthesis of metabolically active proteins [10,13]. Similarly, *kiwicha* germinated at 22 °C for 72 h, increasing from 15.40 to 23.70 g·100 g^−1^ (d.b.) [10], while amaranth germinated under optimized conditions, reached 18.21% compared with 13.00% in the ungerminated grain [15]. It is important to note that part of the increase observed in *kañiwa* reflects a concentration effect resulting from dry matter losses during respiration, particularly through the consumption of carbohydrates as energy sources. Consequently, the increase in protein content should be interpreted as the combined result of metabolic remodeling and changes in the relative composition of the grain during germination [10,13].

The biochemical basis of this response involves two concurrent processes. The first is the synthesis of enzymes and structural proteins required for seedling development, including proteases, hydrolases, stress-response proteins, and enzymes associated with primary and secondary metabolism [6,10,13,46]. The second is the degradation of storage proteins mediated by endogenous proteases, which release amino acids to support germination and early growth. The significant negative quadratic effect of temperature suggests that these processes are not equally favored across the thermal range. Temperatures near 24 °C appear to maximize the balance between protein synthesis, reserve mobilization, and respiratory losses, whereas temperatures above this threshold may accelerate proteolysis and metabolic consumption more rapidly than the formation of new proteins. Conversely, temperatures below the optimum may limit enzymatic activity and metabolic efficiency. This narrow thermal optimum explains the hill-shaped morphology observed in Figure 8 and identifies temperature as the principal factor controlling protein enrichment during *kañiwa* germination [6,10,13,15].

### 3.8. Simultaneous Optimization by Global Desirability Function and Experimental Validation

Simultaneous optimization of the five response variables using the desirability function yielded an overall desirability of D = 0.845, identifying 96 h and 24 °C as the optimal germination conditions for *kañiwa* (Figure 9). The red vertical lines in Figure 9 correspond to time = +1.4142 (96 h) and temperature = −0.0009 (approximately 24 °C), indicating that the selected optimum represents a compromise among responses with distinct surface morphologies. While TSPC and protein content showed maximum regions near the central temperature level, GABA and the antioxidant capacity responses benefited from prolonged germination time. Thus, 24 °C provided a balanced thermal condition capable of preserving the favorable response of phenolic and protein-related variables while allowing substantial enhancement of GABA, ORAC, and FRAP [14,23,29]. The overall desirability value obtained in the present study was comparable to values reported in related RSM studies with pseudocereals, including quinoa germinated at 20 °C for 42 h (D = 0.890) [14] and bread formulations containing germinated pseudocereals (D = 0.704) [18].

The predicted values at the optimum point (96 h, 24 °C), the experimental validation values, and the relative deviations between predicted and observed responses are presented in Table 6. The optimized germination condition promoted substantial increases in all response variables compared with ungerminated *kañiwa*, confirming the effectiveness of the multi-response optimization strategy. The relative deviations between predicted and experimental values varied according to the response evaluated.

The valley-shaped morphology of the ORAC and FRAP surfaces does not imply an optimization direction opposite to that of TSPC. A surface with positive quadratic coefficients attains its maxima at the boundaries of the experimental domain; within the domain explored, those maxima were located in the long-germination-time region, ORAC reached 52,655.7 µmol TE·100 g^−1^ at 96 h and 24 °C (run 6) and FRAP reached 1514.4 µmol TE·100 g^−1^ at 85.5 h and 19.7 °C (run 2), coinciding with the direction of the selected optimum. The compromise imposed by the simultaneous optimization concerned exclusively TSPC and protein content, whose concave surfaces present interior maxima near 60 h, against GABA, ORAC and FRAP, which increase toward the axial boundary at 96 h. The Derringer–Suich desirability function [37] resolves this divergence by transforming each response independently and combining them as a geometric mean; D = 0.845 explicitly quantifies the degree of compromise. Validation confirmed that no response was effectively sacrificed: the experimental TSPC at the optimum (926.9 mg GAE·100 g^−1^) exceeded the highest value recorded at any design point (808.9 mg GAE·100 g^−1^, run 12), confirming that the multi-response optimum outperformed the individual TSPC optimum [14,37].

The relative deviations for the three largest responses—GABA (23.18%), TSPC (26.40%), and FRAP (29.30%)—were all positive: experimental values exceeded predicted ones in every case. A random reproducibility failure would produce deviations of mixed sign; the unidirectional pattern indicates systematic underestimation rather than measurement imprecision, with two complementary structural causes. The first is statistical: the desirability function placed the optimum at the axial boundary of the time axis (x_1_ = +1.4142; 96 h), where prediction variance in a rotatable CCD is maximal, and the GABA model retained only linear and interaction terms with no stationary point, so it necessarily underestimates a response that had not plateaued within the experimental range [26,29,30]. In contrast, ORAC (6.50%) and protein content (3.44%) showed close agreement, consistent with their surfaces having interior curvature and lower sensitivity to the boundary effect. The second cause is biological: the CCD was executed in October 2025 and the validation in March 2026, with the seed stock under dry ambient storage throughout the five-month interval. This interval is consistent with a physiological after-ripening period, a process documented in seeds of cereals and pseudocereals during which abscisic acid (ABA) content declines progressively, the ABA:GA ratio shifts toward dormancy release, and the seed undergoes transcriptomic and metabolic reprogramming [54,55,56]. Each of the three affected responses has a documented mechanistic link to this hormonal transition in other species, providing a basis for interpreting the between-cohort discrepancy. For TSPC, the ABA:GA shift has been shown to upregulate the phenylpropanoid and flavonoid biosynthesis pathways in seeds undergoing dormancy release [57]; independently, ABA-maintained dormancy sustains elevated phenolic accumulation—including ferulic and cinnamic acids—as demonstrated in rice seeds [58], and dormant chickpea seed coats contain significantly higher concentrations of gallic, syringic, and sinapic acids than non-dormant counterparts [59]. For GABA, after-ripening has been associated with removal of ABA-mediated suppression of GAD activity: GABA biosynthesis through GAD2 is required for dormancy release, and GABA-T-deficient seeds with impaired GABA catabolism show enhanced dormancy release [60], suggesting that an after-ripened seed lot may activate the GAD/GABA shunt more rapidly upon imbibition than a freshly harvested one. For FRAP, Reactive Oxygen Species (ROS) accumulated during dry grain storage—particularly H_2_O_2_—partially alleviate dormancy in cereals by activating GA signalling and inhibiting GA catabolism [61]; this same ROS signal upregulates the antioxidant glutathione pool and has been reported to increase total phenolic content by 15–20% during postharvest dry storage [62], providing a plausible mechanism for the higher electron-donating capacity measured by FRAP in the March 2026 validation. In *Chenopodium album* (Amaranthaceae, the family of *kañiwa*), dormancy depth varies between harvest cohorts and is progressively released by after-ripening [63], confirming this mechanism operates within the Chenopodiaceae confirming that this mechanism operates within the Chenopodiaceae lineage. The convergence of these three documented pathways—phenylpropanoid upregulation, putative GAD/GABA shunt activation, and ROS-mediated antioxidant enhancement—through the same after-ripening process provides a coherent bibliographic framework for interpreting the unidirectional positive bias, complementing the statistical boundary-effect explanation [57,59,60,61]. The observed validation values are therefore biologically meaningful outcomes consistent with the enhanced germination responsiveness of an after-ripened seed lot, not measurement artifacts. Collectively, these results confirm that the fitted models are adequate for describing response trends and locating the optimum region, but should not be used for absolute point prediction at the boundary of the experimental domain, nor as universal predictors across harvest cohorts with different after-ripening histories [26,29,45].

It should be acknowledged as a methodological limitation that model terms were retained at α = 0.10 rather than the conventional α = 0.05. This decision was justified by the limited residual degrees of freedom available in the CCD, which comprised 12 experimental runs, including four center points. Several quadratic terms, particularly those associated with FRAP (*p* = 0.074–0.091), would not have been retained under the conventional significance threshold and should therefore be interpreted cautiously. The use of α = 0.10 was intended to preserve potentially relevant response-surface structure in a design with constrained degrees of freedom [26,29,30]. Nevertheless, this choice increases uncertainty in the affected terms and limits confidence in point predictions, particularly near the boundaries of the experimental domain. Accordingly, the FRAP and protein-content models should be interpreted primarily as descriptive tools for identifying response trends and the optimal experimental region, rather than as highly precise point-prediction models.

### 3.9. Reducing Sugar Content and Endogenous Enzyme Activities

The germination process profoundly altered carbohydrate metabolism and the endogenous enzymatic profile of *kañiwa* (Table 7). After 96 h of germination under the optimized conditions (24 °C), total reducing sugars increased 375.10%, while free glucose increased 315.40%. Notably, free glucose accounted for approximately 87.00% of the total reducing sugars in the germinated samples, indicating that glucose became the predominant soluble carbohydrate after prolonged germination. Similar increases in soluble sugars and glucose have been reported in germinated quinoa, where transcriptomic analyses demonstrated a coordinated upregulation of starch and sucrose metabolism together with glycolysis and the tricarboxylic acid cycle, reflecting the transition from reserve storage to active metabolism during seed germination [64].

This marked accumulation demonstrates the extensive mobilization of starch reserves promoted by endogenous hydrolases during seed germination and confirms that the optimized germination process efficiently converted reserve carbohydrates into metabolically available sugars [47]. This behavior is consistent with the activation of gibberellin-mediated starch degradation during germination, which promotes the sequential hydrolysis of starch into dextrins, maltose, and glucose through coordinated *α*-amylase, *β*-amylase, and glucoamylase activities, thereby supplying respiratory substrates for embryo development and secondary metabolism [64].

The predominance of glucose among reducing sugars also provides important physiological evidence regarding the germination stage reached after 96 h. During the initial stages of germination, starch hydrolysis is predominantly catalyzed by *α*-amylase, generating dextrins and maltooligosaccharides. Subsequently, glucoamylases hydrolyze these intermediate products into glucose, resulting in the progressive accumulation of free glucose as germination advances. The high glucose concentration measured at the optimized point, therefore, indicates that starch degradation had already progressed to an advanced stage, approaching the complete conversion of starch-derived oligosaccharides into fermentable monosaccharides [65].

Interestingly, despite the marked accumulation of reducing sugars, amylolytic activity decreased dramatically (91.90%), whereas glucoamylase activity, detected in ungerminated seeds, was no longer detectable after germination. At first glance, these results may appear contradictory; however, they are fully compatible with the physiological regulation of starch degradation during advanced germination. The enzymatic activities reported in Table 7 represent residual activities measured after completion of the 96-h germination process rather than the cumulative activity occurring throughout germination. Consequently, low residual activities likely reflect the exhaustion of starch reserves together with the downregulation or inactivation of hydrolytic enzymes once sufficient soluble sugars have accumulated.

One hypothesis reported in the literature involves feedback regulation by glucose; this mechanism was not evaluated in the present study. Elevated intracellular glucose concentrations have been reported to repress the expression of genes encoding starch-degrading enzymes and to reduce *α*-amylase synthesis through sugar-signaling pathways mediated by hexokinase-dependent sensing mechanisms. Such feedback regulation prevents excessive degradation of reserve carbohydrates after the energetic requirements of the growing embryo have been fulfilled. High intracellular glucose concentrations may also contribute to the downregulation of starch-degrading enzymes via sugar-signaling pathways linked to carbon status [65,66]. However, the relative contribution of this regulatory mechanism remains to be elucidated for *kañiwa*. Enzyme activity measured at the end of germination represents the residual catalytic capacity after reserve mobilization rather than the cumulative activity throughout the process. Reviews on cereal germination indicate that *α*-amylase activity generally reaches its maximum during intermediate germination stages, then declines as starch reserves are depleted and seedling metabolism shifts toward reserve utilization and growth [46,67].

Another complementary explanation concerns enzyme turnover during prolonged germination. Hydrolytic enzymes synthesized during the first stages of germination exhibit finite stability and may undergo progressive denaturation, proteolytic degradation or reduced catalytic efficiency after prolonged metabolic activity. Since the optimized germination conditions extended to 96 h, the enzymatic system responsible for starch mobilization had likely already reached its maximum catalytic performance before the final sampling point, resulting in reduced residual activities despite the extensive hydrolysis already accomplished [46].

Unlike amylases, lipase activity increased significantly during germination (130.43%). Lipid mobilization constitutes another essential metabolic process during seed germination, particularly in pseudocereals whose embryos contain appreciable oil reserves. Lipases catalyze the hydrolysis of triacylglycerols into free fatty acids and glycerol, which subsequently enter *β*-oxidation and the glyoxylate cycle, producing acetyl-CoA and metabolic intermediates required for gluconeogenesis and ATP production. Consequently, the increased lipase activity observed in germinated *kañiwa* reflects the activation of reserve lipid mobilization. Similar increases in lipase activity during germination have been described in cereals and pseudocereals, where lipid mobilization complements carbohydrate metabolism through β-oxidation and the glyoxylate cycle, supplying additional carbon skeletons and ATP required for seedling establishment [65,68].

Protease activity was not detected in either ungerminated or germinated samples under the analytical conditions employed (Table 7). This result must be interpreted as an activity below the detection limit of the assay and not as an absence of proteolysis, which is biochemically incompatible with the magnitude of compositional changes observed during germination. Three analytical factors account for the absence of a detectable signal. First, the assay was conducted at a single pH (6.0), whereas seed proteases encompass multiple families with widely separated optima: aspartic and cysteine proteases, which are the dominant classes during cereal and pseudocereal germination, exhibit optimal activity between pH 3.5 and 4.5, while serine and metalloproteases are active above pH 7.0 [69,70,71]. A single assay pH of 6.0 falls between these optima and does not recover the full proteolytic complement. Second, casein is a heterologous substrate with limited affinity for the endopeptidases responsible for storage protein mobilization, which act preferentially on native seed globulins and albumins [72]. Third, the detection endpoint, TCA-soluble tyrosine equivalents quantified at 280 nm, is considerably less sensitive than chromogenic or fluorogenic substrates, and endogenous protease inhibitors present in the crude extract may further suppress the measurable signal [71]. Indirect evidence indicates that proteolysis was active during germination: the 1710.30% increase in GABA requires a sustained supply of free glutamate, which in the germinating seed derives from the hydrolysis of storage proteins through the glutamate decarboxylase pathway. Characterizing the proteolytic system of *kañiwa* using pH-profile assays and fluorogenic substrates constitutes a direct continuation of this work.

Four limitations of this study should be considered. First, the fitted models are empirical approximations whose prediction variance increases toward the boundaries of the experimental domain; the optimum reported must be understood as the optimum within the domain investigated and not as the true biological optimum, particularly for GABA, whose surface remained monotonically ascending at the boundary. Second, the antioxidant capacity was evaluated exclusively through global spectrophotometric assays (TSPC, ORAC and FRAP), which do not identify the individual phenolic compounds contributing to the observed responses; in the absence of chromatographic characterization, the biochemical interpretation of the antioxidant changes must be regarded as provisional. Third, the activities of GAD, PAL and PPO were not determined; the mechanisms invoked to explain the accumulation of GABA and phenolic compounds are therefore literature-supported hypotheses and not experimentally demonstrated pathways. Fourth, the use of a single extraction buffer (pH 5.0) may have contributed to the under-recovery of serine-type proteases, which is acknowledged as a limitation of the enzymatic characterization (Section 2.14.1).

The practical implementation of the optimized conditions necessitates consideration of three process constraints. Sustaining a temperature of 24 °C over 96 h in the Andean highland region, where *kañiwa* is cultivated, entails a heating requirement and consequently incurs site-specific thermal costs. The microbiological regulation of a humid substrate maintained at 24 °C for the same duration demands specific validation at an industrial scale, beyond the disinfection protocol outlined in Section 2.3. A residence time of 96 h immobilizes working capital and favors a staggered-batch or continuous operational configuration over single-batch processing. The downstream process sequence, including disinfection, imbibition, germination, convective drying, and milling, is compatible with conventional sprouting equipment. Previous research conducted by our group has demonstrated the successful integration of sprouted Andean pseudocereals into bread [18] and extruded products [20]. No techno-economic analysis was conducted in this study; the quantification of production costs and microbiological validation at an industrial scale are essential preliminary steps prior to industrial deployment.

Overall, the combined biochemical evidence demonstrates that the optimized germination process induced extensive mobilization of reserve carbohydrates, resulting in the accumulation of glucose and other reducing sugars while simultaneously reducing residual starch-hydrolyzing enzyme activities after substrate depletion. Concurrent activation of lipases indicates coordinated utilization of both carbohydrate and lipid reserves to sustain seed metabolism. These findings provide indirect evidence consistent with for the enhanced accumulation of GABA, phenolic compounds, and antioxidant capacity observed under the optimized germination conditions, suggesting that controlled germination redirects primary metabolism toward the biosynthesis of nutritionally relevant compounds in *kañiwa*.

## 4. Conclusions

This study successfully optimized the controlled germination conditions of *kañiwa* cv. *Cupi* through a rotatable central composite design combined with the Derringer–Suich desirability function, enabling the simultaneous enhancement of GABA, TSPC, antioxidant capacity (ORAC and FRAP), and protein content. The five second-order polynomial models were statistically significant and showed non-significant lack of fit, confirming the suitability of response surface methodology for describing and optimizing the biochemical responses of germinated *kañiwa*. These models are suitable for identifying optimization trends and locating the region of the optimum within the experimental domain investigated, rather than for accurate point prediction at or beyond its boundaries. The optimal conditions (96 h; 24 °C; D = 0.845) produced substantial experimental increases in all evaluated responses relative to ungerminated seeds. Among these, GABA showed the most pronounced enhancement, increasing 1710.30% to reach 26.12 mg GABA·100 g^−1^, supported by the availability of 16.91 g·100 g^−1^ of glutamic acid as the endogenous precursor of the GAD pathway; to the best of our knowledge, this is the first report of GABA accumulation in *kañiwa* under experimentally optimized germination conditions. Complementary biochemical analyses confirmed that the optimized process induced extensive carbohydrate mobilization, with amylolytic activity decreasing 91.90%, glucoamylase activity becoming undetectable, and lipase activity increasing 130.43%, demonstrating coordinated utilization of carbohydrate and lipid reserves throughout germination. These results establish controlled germination as an effective and sustainable bioprocessing strategy for the valorization of *kañiwa* as a gluten-free functional ingredient, contributing to the development of value-added foods from underutilized Andean crops and providing a scientific and technological foundation for future studies on pilot-scale production, bioaccessibility and incorporation into functional food formulations. Future work should further validate these findings through targeted metabolomic and chromatographic approaches to identify the specific compounds responsible for the observed antioxidant responses.

## Figures and Tables

**Table 1:** Coded and real levels of the independent variables.

Independent Variable	Symbol	−1.41	−1	0	1	1.41
Germination time (h)	X_1_	24.0	34.5	60.0	85.5	96.0
Germination temperature (°C)	X_2_	18.0	19.7	24.0	28.3	30.0

**Table 2:** Central Composite Design matrix for optimization of *kañiwa* controlled germination.

Points	Trials	Coded Levels		Real Levels	
		x_1_	x_2_	X_1_ (h)	X_2_ (°C)
Factorial	1	−1	−1	34.5	19.7
	2	1	−1	85.5	19.7
	3	−1	1	34.5	28.3
	4	1	1	85.5	28.3
Axial	5	−1.41	0	24	24
	6	1.41	0	96	24
	7	0	−1.41	60	18
	8	0	1.41	60	30
Central	9	0	0	60	24
	10	0	0	60	24
	11	0	0	60	24
	12	0	0	60	24

**Table 3:** Experimental values of response variables obtained using the Central Composite Design to optimize *kañiwa* controlled germination conditions.

Trials	GABA (mg GABA·100 g^−1^) *	TSPC (mg GAE·100 g^−1^) ^#^	ORAC (µmol TE·100 g^−1^) ^#^	FRAP (µmol TE·100 g^−1^) ^#^	Protein Content (%) ^#^
1	9.96 ± 0.055	592.2 ± 10.50	33,323.3 ± 1244.8	937.4 ± 18.57	12.51 ± 0.09
2	11.50 ± 0.185	753.7 ± 11.60	41,057.7 ± 1409.5	1514.4 ± 55.38	14.21 ± 0.06
3	9.29 ± 0.508	689.2 ± 4.21	42,720.9 ± 431.3	1230.7 ± 45.64	13.40 ± 0.11
4	23.38 ± 0.060	704.0 ± 9.63	44,824.5 ± 580.2	1484.2 ± 69.29	14.82 ± 0.08
5	6.41 ± 0.206	557.1 ± 3.24	28,303.0 ± 1172.7	913.4 ± 5.01	12.62 ± 0.26
6	22.58 ± 0.178	702.9 ± 3.69	52,655.7 ± 475.7	1451.5 ± 45.37	15.40 ± 0.11
7	6.44 ± 0.314	590.0 ± 7.60	41,380.6 ± 1231.8	1166.1 ± 15.29	13.35 ± 0.22
8	19.42 ± 0.358	606.4 ± 4.86	37,393.2 ± 1247.4	1147.8 ± 34.57	11.53 ± 0.60
9	10.99 ± 0.246	799.6 ± 4.50	32,687.2 ± 798.6	1147.9 ± 28.48	16.54 ± 0.19
10	10.43 ± 0.194	760.9 ± 5.35	21,793.2 ± 1732.5	851.1 ± 34.31	14.83 ± 0.44
11	13.77 ± 0.321	784.9 ± 9.27	18,522.9 ± 426.3	960.2 ± 7.93	16.54 ± 0.10
12	14.73 ± 0.233	808.9 ± 3.51	14,908.5 ± 893.2	890.6 ± 23.12	16.55 ± 0.16
Ungerminated	1.44 ± 0.003	426.9 ± 7.70	14,322.0 ± 458.0	572.9 ± 2.80	11.27 ± 0.69

Values expressed as means ± standard deviation. * Means of two repetitions. ^#^ Means of three repetitions. GABA = *γ*-aminobutyric acid; TSPC = total soluble phenolic compounds; GAE = gallic acid equivalent; ORAC = oxygen radical absorbance capacity; FRAP = ferric reducing antioxidant power; TE = Trolox equivalent. Data shown on a dry basis. The statistical significance of the effects of germination time and temperature on each response was evaluated through the analysis of variance of the fitted regression models (Table 4), which is the significance analysis corresponding to a response surface design.

**Table 4:** Analysis of variance (ANOVA) and goodness-of-fit statistics of the second-order polynomial models for the five response variables of the Central Composite Design.

Source of Variation	GABA (mg GABA·100 g^−1^)	TSPC (mg GAE·100 g^−1^)	ORAC (µmol TE·100 g^−1^)	FRAP (µmol TE·100 g^−1^)	Protein Content (%)
Model	*F* = 27.77	*F* = 12.49	*F* = 11.19	*F* = 11.58	*F* = 16.04
Time (L)	*F* = 42.28; *p =* 0.007	*F* = 41.72; *p =* 0.008	*F* = 4.16; *p =* 0.037	*F* = 18.27; *p =* 0.024	*F* = 8.49; *p =* 0.063
Time (Q)	ns	*F* = 55.49; *p =* 0.005	*F* = 9.58; *p =* 0.005	*F* = 7.29; *p =* 0.074	*F* = 7.43; *p =* 0.072
Temperature (L)	*F* = 24.97; *p =* 0.015	ns	ns	ns	ns
Temperature (Q)	ns	*F* = 87.78; *p =* 0.003	*F* = 8.50; *p =* 0.007	*F* = 6.03; *p =* 0.091	*F* = 25.48; *p =* 0.015
Time × Temperature (1L × 2L)	*F* = 9.01; *p =* 0.058	*F* = 12.30; *p =* 0.039	ns	ns	ns
Lack of fit	*F* = 0.860; *p* = 0.588	*F* = 6.16; *p* = 0.084	*F* = 0.460; *p* = 0.790	*F* = 0.850; *p* = 0.592	*F* = 0.780; *p* = 0.625
R^2^	0.912	0.863	0.784	0.802	0.843
R^2^ adjusted	0.880	0.785	0.703	0.727	0.785
*p*-value	<0.001	0.003	0.003	0.003	0.001
CV%	15.77	5.52	18.30	10.90	5.97

ns = non-significant effect, excluded from the final model. L = linear effect; Q = quadratic effect; GABA = *γ*-aminobutyric acid; TSPC = total soluble phenolic compounds; GAE = gallic acid equivalent; ORAC = oxygen radical absorbance capacity; FRAP = ferric reducing antioxidant power; TE = Trolox equivalent.

**Table 5:** Regression coefficients of the final second-order polynomial models fitted for GABA, TSPC, ORAC, FRAP, and protein content as a function of germination time (X_1_) and temperature (X_2_) in *kañiwa*.

Coefficient	Term	GABA (mg GABA·100 g^−1^)	TSPC (mg GAE·100 g^−1^)	ORAC (µmol TE·100 g^−1^)	FRAP (µmol TE·100 g^−1^)	Protein (%)
*β*_0_	Intercept	13.24	788.6	21,978.0	962.5	16.11
*β*_1_	X_1_ (L)	4.81	47.80	5534.2	198.9	0.880
*β*_2_	X_2_ (L)	3.70	ns	ns	ns	ns
*β*_11_	X_1_^2^ (Q)	ns	−61.63	9387.3	140.5	−0.920
*β*_22_	X_2_^2^ (Q)	ns	−77.51	8841.8	127.7	−1.71
*β*_12_	X_1_X_2_	3.14	−36.70	ns	ns	ns

ns = non-significant effect, excluded from the final model by backward elimination (*p* > 0.10). L = linear effect; Q = quadratic effect; GABA = *γ*-aminobutyric acid; TSPC = total soluble phenolic compounds; GAE = gallic acid equivalent; ORAC = oxygen radical absorbance capacity; FRAP = ferric reducing antioxidant power; TE = Trolox equivalent.

**Table 6:** Predicted values, experimental validation values, relative deviations, and percentage increases compared with ungerminated *kañiwa* for the five response variables under the optimized germination conditions (96 h, 24 °C; D = 0.845).

Dependent Variable	Predicted Value	Experimental Value	Relative Deviation (%)	Increase vs. Ungerminated Seeds (%)
GABA (mg GABA·100 g^−1^)	21.21	26.12	23.18	1710.30
TSPC (mg GAE·100 g^−1^)	732.9	926.9	26.40	117.10
ORAC (µmol TE·100 g^−1^)	48,575.7	51,756.0	6.55	261.40
FRAP (µmol TE·100 g^−1^)	1524.8	1971.2	29.30	244.10
Protein content (%)	15.51	16.05	3.44	42.34

**Table 7:** Effects of optimized germination on carbohydrate mobilization and endogenous hydrolytic enzyme activities in *kañiwa*.

Sample	Ungerminated *kañiwa* Seeds	Optimal Point Germinated *kañiwa*	*p*-Value
Total reducing sugars *	13.59 ± 0.450	64.56 ± 2.87	<0.001
Free glucose *	13.52 ± 0.450	56.16 ± 0.820	<0.001
Amylolytic activity ^#^	44.46 ± 0.110	3.60 ± 0.060	<0.001
Glucoamylase activity ^#^	22.84 ± 2.05	nd	—
Lipase activity ^#^	0.023 ± 0.002	0.053 ± 0.002	<0.001
Protease activity ^#^	nd	nd	—

Values reported as means of three replicates ± standard deviation; * Values expressed as mg of glucose·g^−1^ of sample (d.b.); ^#^ Values expressed as U·g^−1^ of sample (d.b.); Different values between ungerminated and germinated samples were compared using Student’s *t*-test (*p* < 0.05); nd = not detected, i.e., below the detection limit of the assay under the conditions employed (1% *w*/*v* casein in 100 mM sodium acetate buffer, pH 6.0; 50 °C; detection of TCA-soluble tyrosine equivalents at 280 nm).

## Data Availability

Data is contained within the article.

## Abbreviations

The following abbreviations are used in this manuscript:

AAPH	2,2′-Azobis(2-amidinopropane) dihydrochloride
ABA	Abscisic acid
ANOVA	Analysis of variance
AOAC	Association of Official Analytical Chemists
ATP	Adenosine triphosphate
AUC	Area under the curve
CCD	Central Composite Design
CoA	Coenzyme A
CV%	Coefficient of variation
d.b.	Dry basis
DAD	Diode-array detector
DF	Dilution factor
DNS	3,5-Dinitrosalicylic acid
DPPH	2,2-Diphenyl-1-picrylhydrazyl
EDTA	Ethylenediaminetetraacetic acid
EEA	Estación Experimental Agraria
FRAP	Ferric reducing antioxidant power
GA	Gibberellin
GABA	*γ*-Aminobutyric acid
GABA-T	GABA-transaminase
GAD	Glutamate decarboxylase
GAE	Gallic acid equivalents
GOPOD	Glucose oxidase–peroxidase
HPLC	High-performance liquid chromatography
IGN	Instituto Geográfico Nacional del Perú
INIA	Instituto Nacional de Innovación Agraria
LoF	Lack of fit
m.a.s.l.	Metres above sea level
nd	Not detected
ORAC	Oxygen radical absorbance capacity
PAL	Phenylalanine ammonia-lyase
PLP	Pyridoxal 5′-phosphate
PPO	Polyphenol oxidase
R^2^	Coefficient of determination
R^2^adj	Adjusted coefficient of determination
RH	Relative humidity
ROO•	Peroxyl radical
ROS	Reactive oxygen species
RP-HPLC	Reverse-phase high-performance liquid chromatography
RSM	Response Surface Methodology
TCA	Trichloroacetic acid
TE	Trolox equivalents
TEAC	Trolox equivalent antioxidant capacity
TPTZ	2,4,6-Tri(2-pyridyl)-s-triazine
TSPC	Total soluble phenolic compounds
UTM	Universal Transverse Mercator
WGS	World Geodetic System
